# *Staphylococcus epidermidis* biofilms undergo metabolic and matrix remodeling under nitrosative stress

**DOI:** 10.3389/fcimb.2023.1200923

**Published:** 2023-07-04

**Authors:** Ana S. Oliveira, Lígia M. Saraiva, Sandra M. Carvalho

**Affiliations:** Instituto de Tecnologia Química e Biológica António Xavier, Universidade Nova de Lisboa (ITQB NOVA), Oeiras, Portugal

**Keywords:** *Staphylococcus epidermidis*, biofilm metabolism, nuclear magnetic resonance (NMR), confocal laser scanning microscopy (CLSM), nitrosative stress

## Abstract

*Staphylococcus epidermidis* is a commensal skin bacterium that forms host- and antibiotic-resistant biofilms that are a major cause of implant-associated infections. Most research has focused on studying the responses to host-imposed stresses on planktonic bacteria. In this work, we addressed the open question of how *S. epidermidis* thrives on toxic concentrations of nitric oxide (NO) produced by host innate immune cells during biofilm assembly. We analyzed alterations of gene expression, metabolism, and matrix structure of biofilms of two clinical isolates of *S. epidermidis*, namely, 1457 and RP62A, formed under NO stress conditions. In both strains, NO lowers the amount of biofilm mass and causes increased production of lactate and decreased acetate excretion from biofilm glucose metabolism. Transcriptional analysis revealed that NO induces *icaA*, which is directly involved in polysaccharide intercellular adhesion (PIA) production, and genes encoding proteins of the amino sugar pathway (*glmM* and *glmU*) that link glycolysis to PIA synthesis. However, the strains seem to have distinct regulatory mechanisms to boost lactate production, as NO causes a substantial upregulation of *ldh* gene in strain RP62A but not in strain 1457. The analysis of the matrix components of the staphylococcal biofilms, assessed by confocal laser scanning microscopy (CLSM), showed that NO stimulates PIA and protein production and interferes with biofilm structure in a strain-dependent manner, but independently of the Ldh level. Thus, NO resistance is attained by remodeling the staphylococcal matrix architecture and adaptation of main metabolic processes, likely providing *in vivo* fitness of *S. epidermidis* biofilms contacting NO-proficient macrophages.

## 1 Introduction

The biofilm lifecycle is a dynamic process starting with the binding of planktonic cells to (a)biotic surfaces, promoted by several bacterial proteinaceous adherence factors (Otto, 2018). The attached cells proliferate and secrete major biofilm matrix polymeric substances, namely, polysaccharide intercellular adhesin (PIA), also known as poly-*N*-acetylglucosamine (PNAG) exopolysaccharide, synthesized by proteins encoded by the *icaADBC* operon, extracellular DNA (eDNA), originated from cell lysis, and proteins, all contributing to cell accumulation via adhesion and formation of mature biofilms. The subsequent dispersion of mature biofilm cells and spread to other sites in the body trigger the initiation of a new cycle of biofilm formation (Rohde et al., 2010; Otto, 2018; Nguyen et al., 2020). Furthermore, the success in the assembling of planktonic microbial cells conducive to biofilm formation depends on their ability to resist and evade the effectors of the host immune system (Le et al., 2018). In particular, cells of the innate immune system, which is the first to be activated when infection occurs, release chemicals that impose chemical stresses on invading pathogens (Dapunt et al., 2016). M1 macrophages are one of the main cells of this system that infiltrate the tissue/implant interface (Martin and García, 2021) and promote bacterial clearance via the nitric oxide (NO) produced by the inducible nitric oxide synthase (iNOS) enzyme (Carvalho et al., 2022). Several studies performed in planktonic state showed that bacterial resistance is dependent on metabolic remodeling and detoxification/repair systems to overcome NO inhibition of main bacterial components, such as respiratory enzymes (e.g., heme–copper oxygen reductases) and iron–sulfur proteins (e.g., tricarboxylic acid (TCA) cycle aconitase and fumarase, and dihydroxyacid dehydratases) (Justino et al., 2007; Nobre et al., 2014; Carvalho et al., 2017; Carvalho et al., 2021). Mature biofilms (which, depending on the bacteria and growth conditions, are formed between 24 h and several days) (Wu et al., 2014; Post et al., 2017; Chen et al., 2020; Haidari et al., 2021) form hypoxic microenvironments, where the low oxygen availability impairs iNOS activity and promotes polarization of M1 NO-producing to M2 anti-inflammatory macrophages, thus reducing the amount of NO that is produced (Christner et al., 2010; Schommer et al., 2011; Thurlow et al., 2011; Yamada and Kielian, 2019). In most bacterial species, including *Staphylococcus epidermidis*, low NO concentrations (in the picomolar to nanomolar range) elicit the dispersion of bacteria from mature biofilms (De La Fuente-Núñez et al., 2013; Barraud et al., 2014; Arora et al., 2015). However, there are also a few cases of enhancement of biofilm formation in bacteria (e.g., *Vibrio harveyi*, *Staphylococcus aureus*) triggered by the addition of low NO amounts (Arora et al., 2015). In *Neisseria gonorrhoeae* and *Shewanella oneidensis*, the NO effect is oxygen-dependent, leading to biofilm dispersal under aerobiosis and enhancement of biofilm formation under anaerobic conditions (Arora et al., 2015). In *Pseudomonas aeruginosa*, endogenous NO produced from the activity of nitrite reductase (NirS) was shown to stimulate biofilm formation (De La Fuente-Núñez et al., 2013).

**Figure 1:**
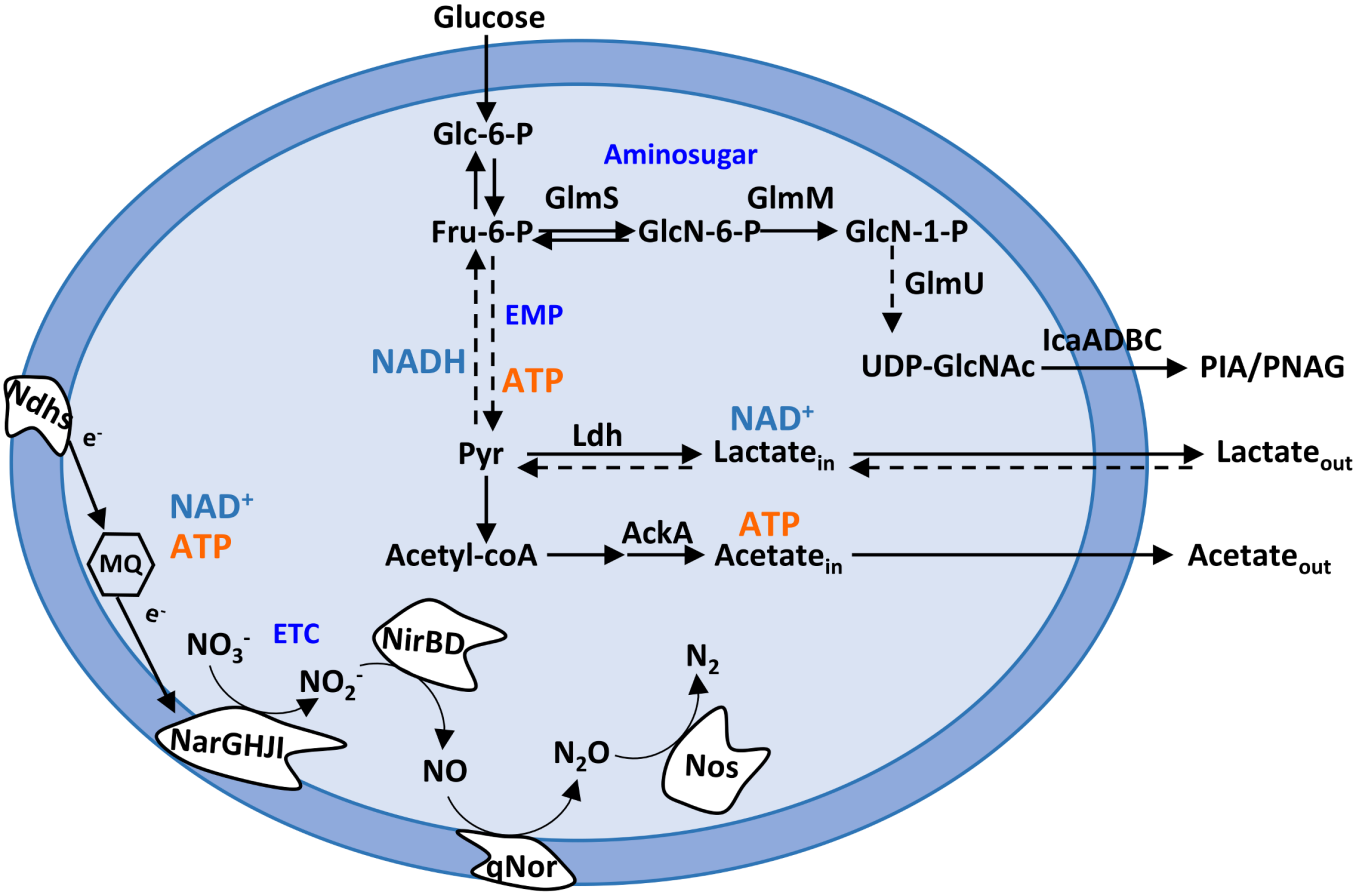
*Staphylococcus epidermidis* metabolic pathways relevant to this study. Schematic representation of the Embden–Meyerhof–Parnas (EMP; glycolytic) pathway, amino sugar pathway (GlmS, GlmM, and GlmU), pyruvate metabolism, and nitrate and nitrite respiration. AckA, acetate kinase; ETC, electron transport chain; Fru-6-P, fructose-6-phosphate; Glc-6-P, glucose-6-phosphate; GlcN-6-P, glucosamine-6-phosphate; GlcN-1-P, glucosamine-1-phosphate; GlmS, glutamine fructose-6-phosphate transaminase; GlmM, phosphoglucosamine mutase; GlmU, glucosamine 1-phosphate *N*-acetyltransferase; IcaA, poly-beta-1,6-*N*-acetyl-d-glucosamine synthase; Ldh, lactate dehydrogenase; MQ, menaquinone; Ndhs, NADH dehydrogenases; NarGHJI, nitrate reductase; NirBD, nitrite reductase; Nos, nitrous oxide reductase; NO_3_^−^, nitrate; NO_2_^−^, nitrite; NO, nitric oxide; N_2_O, nitrous oxide; N_2_, dinitrogen; Pyr, pyruvate; PIA, polysaccharide intercellular adhesin; PNAG, poly-*N*-acetylglucosamine; qNor, quinol-dependent nitric oxide reductase; UDP-GlcNAc, UDP-*N*-acetylglucosamine.

*S. epidermidis* is a ubiquitous coagulase-negative (CoN) human skin commensal involved in skin homeostasis and repair and out competition of opportunistic pathogens (Severn and Horswill, 2023). However, *S. epidermidis* is also an opportunistic pathogen that forms robust biofilms in host tissues and medical devices (Severn and Horswill, 2023). As such, *S. epidermidis* is a leading causative agent of implant biofilm-associated infections, namely, prosthetic joint infections (approximately 30%–43%), catheter-related bloodstream infections (approximately 30%–40%), and prosthetic valve endocarditis, pacemaker, and neonatal sepsis infections (approximately 13%) (Skovdal et al., 2022; Severn and Horswill, 2023). These diseases are very difficult to treat due to the widespread multidrug-resistant lineages of *S. epidermidis* (MRSE) (Lee et al., 2018). *S. epidermidis* is a heterotrophic facultative anaerobe that can use aerobic respiration, anaerobic respiration, or fermentation to produce energy from environmental carbon sources (Pedroza-Davila et al., 2020; Martínez-García et al., 2021; Calvo et al., 2022). Hypoxia and anoxic environments provide *sine qua non* conditions for increased production of *S. epidermidis* biofilm mass (Pedroza-Davila et al., 2020; Martínez-García et al., 2021; Calvo et al., 2022). Oxygen diffusion studies have shown that rapid oxygen depletion occurs within the biofilm (from 4 to 6 h) (Cotter et al., 2009; Simkins et al., 2018). Moreover, transcriptomic data on *S. epidermidis* biofilms (formed after 24 h) revealed downregulation of genes encoding proteins related to aerobic energy production and upregulation of anaerobic/fermentative gene encoding enzymes in relation to planktonic and non-adherent cells (Yao et al., 2005). Glycolysis or the Embden–Meyerhof–Parnas pathway does not require oxygen for the catabolism of carbon sources and is linked to the biosynthesis of PIA from UDP-*N*-acetylglucosamine via the glycolytic fructose-6-phosphate and the amino sugar metabolic pathway, encoded by *glmS*, *glmM*, and *glmU* genes (Sadykov et al., 2008; Sadykov et al., 2011) (**Figure 1**). In planktonically grown *S. epidermidis*, production of PIA increases when glucose is highly available and redirected to amino sugar metabolism (Vuong et al., 2005; Sadykov et al., 2011). This redirection of glucose metabolism is associated with a decrease in the TCA cycle activity under aerobic conditions and is controlled by the metabolic regulator catabolite control protein A (CcpA) (Vuong et al., 2005; Sadykov et al., 2011). However, how *S. epidermidis* adapts its metabolism in the presence of nitric oxide while forming biofilms and whether NO causes adaptive changes in the *S. epidermidis* biofilm matrix remain unknown.

In this work, biofilms of two model organisms of *S. epidermidis*, namely, strains 1457 and RP62A, were exposed to NO stress to analyze its impact on growth and metabolism. The choice of the strains was based on the following: 1457 and RP62A are clinical isolates from major catheter-associated infections and are strains widely used in studies of staphylococcal biofilm formation that produce PIA-dependent biofilms (Hoang et al., 2019; Foster, 2020; Ortega-Peña et al., 2020; Calvo et al., 2022; Joubert et al., 2022; Sung et al., 2022). We observed that NO interferes with the matrix composition and structure of the *S. epidermidis* biofilms and alters the metabolism in a strain-dependent manner. The results broaden our understanding of how biofilm development of pathogenic bacteria copes with the antimicrobial activity of NO produced by cells of the mammalian innate immune system.

## 2 Results

### 2.1 Biofilms of *S. epidermidis* produce more lactate when exposed to nitric oxide

**Figure 2:**
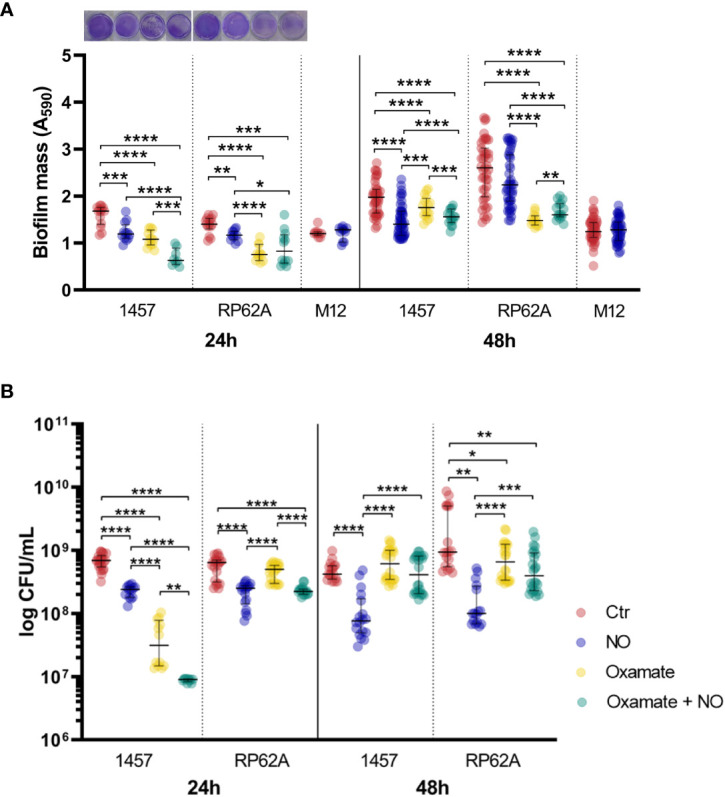
Mass and viability of *Staphylococcus epidermidis* biofilms exposed to NO and oxamate. **(A)** Amount of biofilm formed by *S. epidermidis* 1457, RP62A, and M12 strains grown for 24 and 48 h in high-glucose DMEM/FBS, in the absence (Ctr, red dots) and presence of 1 mM of NO (blue dots). Biofilm amounts of strains 1457 and RP62A were also assessed in the presence of 5 mM of oxamate (yellow dots) and oxamate+NO (green dots). Biofilm mass was determined via the crystal violet assay by measuring absorbance at 590 nm. Representative staining of the 24-h biofilms with crystal violet in well plates is shown in the graph. **(B)** Viable biofilm-encased cells determined by CFU counting. Scattered symbols represent individual measurements, and horizontal lines indicate median values and interquartile range; *n* ≥ 14 for Ctr, *n* ≥ 12 for NO and oxamate conditions, and *n* ≥ 9 for oxamate+NO. Comparisons were performed using Welch’s t-tests. Asterisks represent statistically significant differences (****, *p* ≤ 0.0001; ***, *p* ≤ 0.001; **, *p* ≤ 0.01; *, *p* ≤ 0.05). DMEM, Dulbecco’s modified Eagle medium; FBS, fetal bovine serum; CFU, colony-forming unit.

During the first stages of biofilm assembly in implant medical devices, bacterial cells are exposed to toxic micromolar concentrations of NO produced by cells of innate immunity (Arora et al., 2015; Rinaldo et al., 2018). To elucidate how *S. epidermidis* copes metabolically with this stress, we determined the end-products excreted from the glucose metabolism (**Figure 1**) of cells forming biofilms under NO stress. We used strains 1457 and RP62A, which are described as strong biofilm producers (**Table S1**), grown under static conditions in 24-well plates in high glucose- and fetal bovine serum (FBS)-containing Dulbecco’s modified Eagle medium (DMEM) in the presence of the NO releaser DETANONOate. We analyzed the supernatants of biofilms formed after 24 and 48 h by ^1^H-NMR to evaluate substrate consumption and metabolic end-products. The time considered for biofilm formation, i.e., 24 and 48 h, was chosen based on the following rationale: biofilms at 24 h allow observation of the end-products that are accumulated during early-stage biofilm formation, and at 48 h, a mature biofilm is present. DETANONOate is a slow NO donor releaser with a half-life of 20 h, at 37°C and pH 7, which was used at 1 mM concentration so that small micromolar quantities of NO are released over time of biofilm development. We started by evaluating the NO release from DETANONOate through the determination of the amount of nitrite accumulated in the supernatant of biofilms formed after 24 and 48 h (**Figure S1**). For simplicity, we hereafter designate NO stress as the stress generated by adding DETANONOate. Our data show that DETANONOate (1 mM) leads to a significant decrease in the amount of biofilm mass formed by both strains of *S. epidermidis* (**Figures 2A**, **S2**). We observed that NO also interferes with the viability of biofilm-encased cells (**Figure 2B**). For strain 1457, in mean terms, a 2.5-fold lower biofilm viability was always observed comparatively to the control without NO, i.e., independently of the age of the biofilm. In strain RP62A, the NO-exposed biofilm-encased cells showed 6- and 9.5-fold lower viabilities than those non-exposed, at 24 and 48 h, respectively (**Figure 2B**).

**Figure 3:**
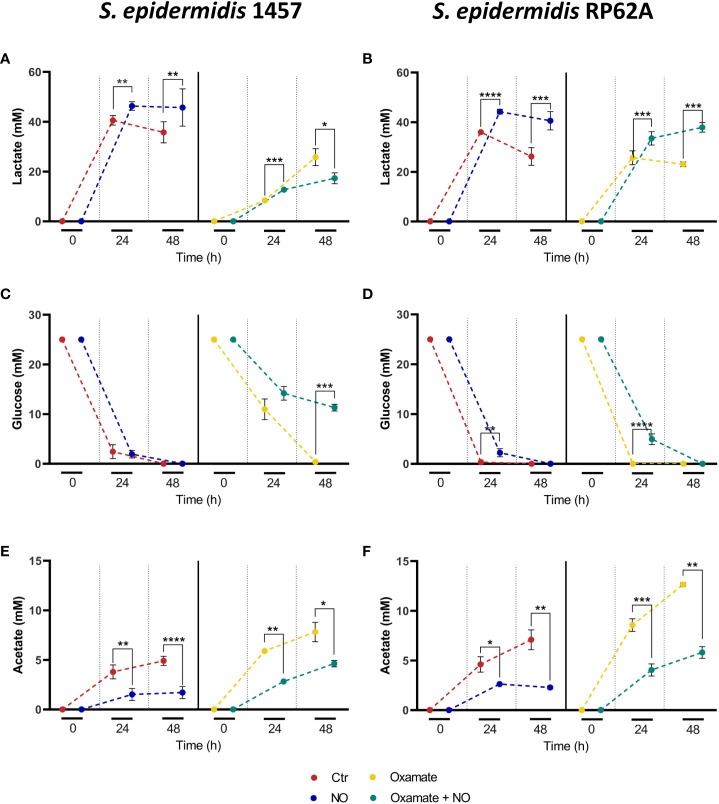
Substrate consumption and major end-products formed by NO- and oxamate-exposed biofilms of strains 1457 and RP62A. Lactate accumulation **(A, B)**, glucose consumption **(C, D),** and acetate accumulation **(E, F)** by biofilms of 1457 **(A, C, E)** and RP62A **(B, D, F)** strains, grown in high-glucose DMEM/FBS, unexposed (Ctr, red) and exposed to 1 mM of NO (blue) and 5 mM of oxamate (yellow) and oxamate+NO (green). Biofilm supernatant samples for substrate and end-product concentration analysis by ^1^H-NMR were harvested at the time of inoculation (0 h) and after 24 and 48 h of biofilm formation. To allow better observation of the differences between the conditions, the points at each hour in each graph were represented with a small shift, which does not indicate measurements at different times. Error bars represent concentrations’ means ± SD (*n* ≥ 6 for 48-h data and *n* ≥ 3 for 24-h data). Comparisons were performed using Welch’s t-tests. Asterisks represent statistically significant data relative to control (****, *p* ≤ 0.0001; ***, *p* ≤ 0.001; **, *p* ≤ 0.01; *, *p* ≤ 0.05).

Metabolically, untreated biofilms of strains 1457 and RP62A exhibited after 24 h a homolactic behavior, with lactate (l-lactate+d-lactate) as the major end-product reaching concentrations of 40 ± 2 and 36 ± 0 mM, which account for approximately 80% and 70% of the glucose consumed, respectively (**Figures 1**, **3A–D**, left panels; **Table 1**). Acetate is also formed but in lower amounts (3–5 mM) (**Figures 1**, **3E, F**, left panels; **Table 1**), together with minor quantities (µM) of formate and succinate (data not shown). It should be noted that *S. epidermidis* encodes two lactate dehydrogenases, the l- and d-lactate dehydrogenases (l-Ldh and d-Ldh), which produce lactate. In this study, the lactate measured is the sum of l-lactate and d-lactate, as they cannot be distinguished by conventional ^1^H-NMR methodology.

**Table 1:** End-products and ATP yields, carbon recovery, and redox balance of biofilms of strains 1457, RP62A, and M12 grown in DMEM/FBS containing 26.3 mM of glucose, untreated (Ctr) and exposed to 1 mM of NO and 5 mM of oxamate and oxamate+NO.

Growth condition	Ctr	NO	Oxamate		Oxamate+NO	
Biofilm age (h)	24	24	24	48	24	48
*S. epidermidis* 1457						
**Product yields**^a^						
Lactate	1.60 ± 0.07	1.89 ± 0.14	0.62 ± 0.07	1.00 ± 0.12	1.06 ± 0.10	1.15 ± 0.11
Acetate	0.14 ± 0.02	0.06 ± 0.02	0.44 ± 0.04	0.30 ± 0.03	0.24 ± 0.03	0.31 ± 0.02
**Glucose consumed (mM)**	25.1 ± 0.4	24.9 ± 0.7	13.5 ± 0.9	25.8 ± 0.4	12.1 ± 1.4	15.0 ± 0.7
**Substrate consumption (%)**	95 ± 2	95 ± 3	51 ± 3	98 ± 2	46 ± 5	57 ± 2
**Carbon recovery/balance**^b^ **%**	92 ± 4	97 ± 7	62 ± 7	73 ± 8	66 ± 7	76 ± 6
**Redox balance**^c^	89 ± 3	94 ± 7	50 ± 6	65 ± 7	55 ± 5	63 ± 6
**ATP yield (mol/mol substrate)**	1.97 ± 0.11	2.01 ± 0.15	1.69 ± 0.18	1.76 ± 0.20	1.55 ± 0.17	1.83 ± 0.14
*S. epidermidis* RP62A						
**Product yields**^a^						
Lactate	1.40 ± 0.02	1.88 ± 0.06	0.91 ± 0.02	NA	1.46 ± 0.09	NA
Acetate	0.18 ± 0.03	0.11 ± 0.00	0.33 ± 0.02	NA	0.18 ± 0.02	NA
**Glucose consumed (mM)**	25.7 ± 0.2	23.4 ± 0.1	26.3 ± 0.0	NA	22.1 ± 1.0	NA
**Substrate consumption (%)**	98 ± 1	89 ± 1	100 ± 0	NA	84 ± 4	NA
**Carbon recovery/balance**^b^ **%**	86 ± 2	100 ± 3	65 ± 2	NA	82 ± 5	NA
**Redox balance**^c^	85 ± 2	94 ± 3	51 ± 1	NA	74 ± 4	NA
**ATP yield (mol/mol substrate)**	1.90 ± 0.06	2.11 ± 0.07	1.62 ± 0.06	NA	1.83 ± 0.13	NA
*S. epidermidis* M12						
**Product yields**^a^						
Lactate	1.88 ± 0.02	1.89 ± 0.13				
Acetate	0.14 ± 0.02	0.09 ± 0.01				
**Glucose consumed (mM)**	25.1 ± 0.3	21.6 ± 0.4				
**Substrate consumption (%)**	96 ± 1	82 ± 2				
**Carbon recovery/balance**^b^ **%**	102 ± 0	99 ± 7				
**Redox balance**^c^	96 ± 0	95 ± 6				
**ATP yield (mol/mol substrate)**	2.18 ± 0.02	2.07 ± 0.14				

DMEM, Dulbecco’s modified Eagle medium; FBS, fetal bovine serum; NA, not applied.

a Product yield of major end-products is calculated as [product]/[consumed glucose].

b Carbon recovery/balance is the percentage of carbon in metabolized glucose that is recovered in the fermentation products.

c Redox balance is the percentage of NAD^+^ in metabolized glucose that is formed alongside fermentation products, whose production involve regeneration of NAD^+^.

NO caused a significant increase in lactate production (~ 20% for both strains), which accounts for approximately 90% and 94% of the glucose consumed in strains 1457 and RP62A, respectively (**Figures 1**, **3A–D**, left panels; **Table 1**), denoting a typically fermentative metabolism. The yield and amount of acetate, which accounts for 7% (strain 1457) and 9% (strain RP62A) of the glucose consumed in untreated biofilms, were significantly decreased by NO by approximately twofold in both strains (**Figures 1**, **3E, F**, left panels; **Table 1**). The formate and succinate, which are present in the control, were formed in negligible amounts in the NO-exposed cells (data not shown). Interestingly, the strains exposed to NO accumulated a compound, which is absent in non-exposed cells, that resonates in the ^1^H-NMR spectrum as a doublet of triplets between 2.8 and 3.2 ppm (**Figure S3**), most probably due to the formation of a nitrosylated compound, that was not possible to identify by two-dimensional NMR.

The synthesis of ATP by fermentation relies on substrate-level phosphorylation. Although NO caused a significant increase in the lactate amount, the ATP yields obtained by substrate-level phosphorylation were only slightly increased in strain RP62A and were not modified in strain 1457 (**Table 1**). These results indicate that the decrease in ATP/acetate production in NO-stressed biofilms was counterbalanced by the increase in NAD^+^/lactate production (**Figures 1**, **3A–F**, left panels; **Table 1**). Moreover, the carbon recovery and redox balance of NO-treated biofilms were 5% and 10%–15% higher than in 1457- and RP62A-untreated biofilms, respectively (**Table 1**), showing deviation of carbon from other biochemical processes to the formation of higher amounts of end-products, i.e., lactate/NAD^+^ (**Figure 1**).

Analysis of the supernatants of biofilms formed after 48 h showed a decrease of the lactate produced when compared to those formed after 24 h, which was independent of the exposure to NO, thus suggesting that lactate is used as a carbon source after glucose is exhausted from the medium (**Figures 3A–D**, left panels). The differences in the end-products profile observed at 24 h between NO-treated and untreated biofilms were maintained at 48 h (**Figures 3A–F**, left panels).

**Figure 4:**
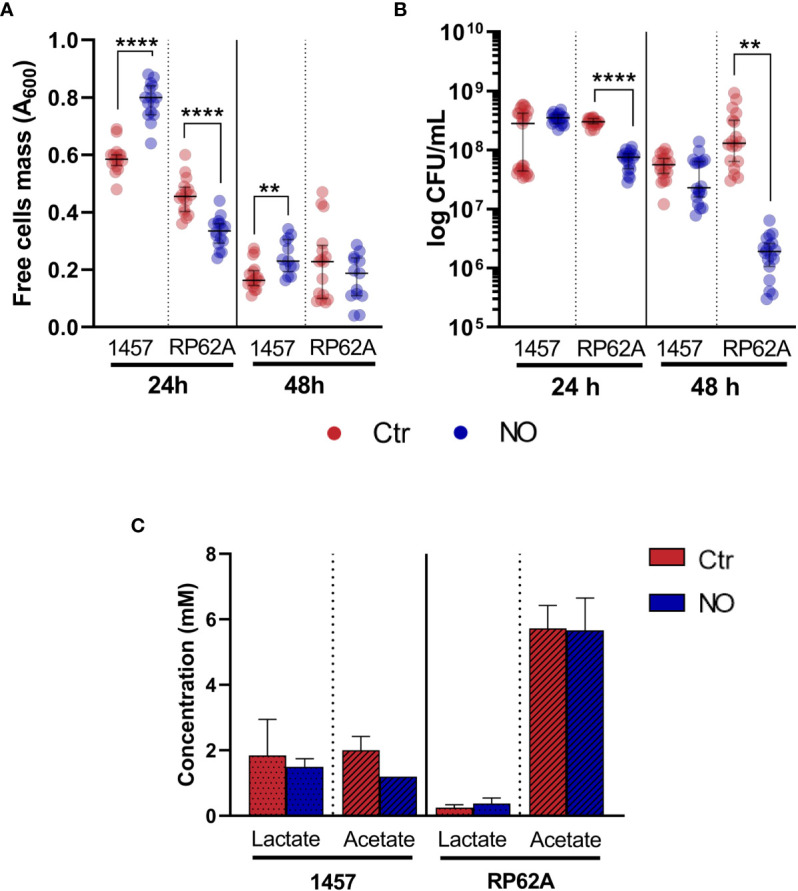
Mass and viability of biofilm-free cells covering *Staphylococcus epidermidis* biofilms exposed to NO and extracellular lactate accumulated by planktonically grown *S. epidermidis* exposed to NO. Mass **(A)** and viability **(B)**, measured by OD_600_ and CFUs, respectively, of free cells recovered from the media covering *S. epidermidis* biofilms of strains 1457 and RP62A grown for 24 and 48 h in high-glucose DMEM/FBS, exposed (blue dots) or not (Ctr, red dots) to 1 mM of NO. Lactate and acetate concentrations **(C)** were determined by ^1^H-NMR in culture supernatants of *S. epidermidis* strains 1457 and RP62A grown in high-glucose DMEM/FBS in planktonic conditions for 24 h in the absence (Ctr, red bar) and presence of 1 mM of NO (blue bar). Scattered symbols represent individual measurements (*n* ≥ 14 and *n* ≥ 18 for free cells mass and viability, respectively), and horizontal lines indicate median values and interquartile range. Error bars represent mean ± SD (*n* = 3). Welch’s t-tests were performed. Asterisks represent statistically significant data relative to control (****, *p* ≤ 0.0001; **, *p* ≤ 0.01). CFUs, colony-forming units; DMEM, Dulbecco’s modified Eagle medium; FBS, fetal bovine serum.

Interestingly, while the biomass (OD_600_) and the number of viable non-adherent cells in the medium covering the biofilms of strain RP62A decrease in the presence of NO, which is an effect that also occurs under planktonic conditions (data not shown), in strain 1457, the free cell biomass increased at 24 h under NO stress (**Figures 4A, B**), suggesting that in this strain, free cell metabolism may contribute to higher lactate yield. Notably, strains 1457 and RP62A grown planktonically, i.e., as free cells, in the same medium used for the biofilms and in the presence of 1 mM of NO did not show higher lactate amounts, indicating that the higher lactate production caused by NO is biofilm-specific (**Figure 4C**). Moreover, the data in strain 1457 suggest that NO promotes the release of cells from the biofilm and/or impairs their attachment to the biofilm, favoring non-adherent planktonic growth of a population of cells.

To investigate whether the effect of NO on lactate production was associated with strong-biofilm-producing strains, *S. epidermidis* 1457-M12, which is a mutant of strain 1457 described as a low PIA and biofilm-producing strain (Mack et al., 2000) (**Table S1**), hereafter designated as M12, was analyzed under NO stress. We observed that strain M12 produced significantly lower biofilm mass than strains 1457 (*p*-value < 0.0001 at 24 h and < 0.01 at 48 h) and RP62A (*p*-value < 0.0001 at 24 h and < 0.0001 at 48 h, Welch’s t-tests) (**Figure 2A**). Moreover, *S. epidermidis* M12 exhibited high lactate yields and a carbon recovery of approximately 100% (**Table 1**), suggesting that the production of lactate occurred at its maximal. Interestingly, NO did not affect the lactate yield and biofilm mass, contrary to what was observed for strains 1457 and RP62A (**Figures 2A**; **S4A–C**, **Table 1**). However, in strain M12, the acetate yield also decreased in NO-treated cells (**Figure S4C**, **Table 1**). Therefore, in strains 1457 and RP62A under NO stress, the higher lactate concentration measured during biofilm formation most likely reflects the NO inhibition of enzymes that act downstream of pyruvate (e.g., pyruvate-formate lyase and pyruvate dehydrogenase) (Richardson et al., 2008), leading to acetate production, and the higher activity of Ldh that deviates carbon to the production of lactate (**Figure 1**).

Strains 1457 and RP62A grown in Tryptic Soy Broth (TSB), a low glucose- and high peptide-containing non-physiological medium with unknown chemical defined composition and widely used in *in vitro* experiments with staphylococci, were exposed to 1 mM of NO. The formation of biofilms with lower mass and viability and with higher lactate production (**Figure S5**) was also observed, which indicates that the effect of NO on the biofilm characteristics is independent of the medium composition.

Thus, we conclude that NO increases lactate production in *S. epidermidis* biofilms, and this increase seems to be characteristic of strongly biofilm-producing strains.

### 2.2 Biofilms of strains 1457 and RP62A reveal different patterns of expression of the lactate dehydrogenase *ldh* gene

**Figure 5:**
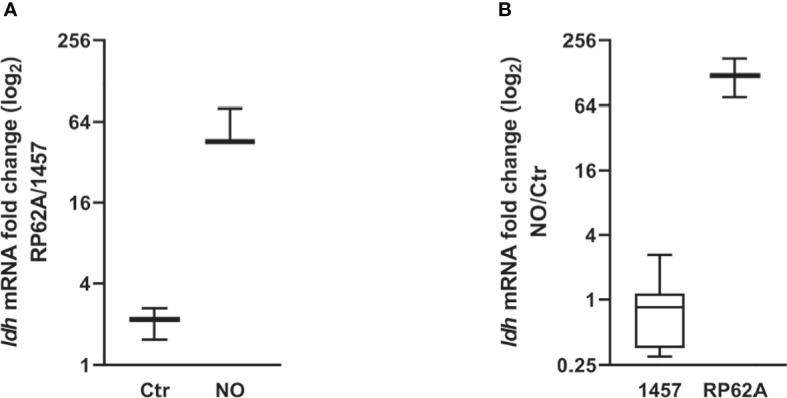
NO-induced *ldh* expression changes in *Staphylococcus epidermidis* 1457 and RP62A biofilms. Log-2-fold changes in gene expression of *ldh* from 24-h biofilms of **(A)** strain RP62A as compared to strain 1457, in the absence and presence of NO, and **(B)** 1457 and RP62A biofilms with NO as compared to control without NO addition. Boxes represent the interval between the 25th and 75th percentiles, and intermediate lines mark medians. Error bars represent minimum and maximum values (*n* ≥ 12). The expression ratios of the genes were normalized relative to the *16S* constitutive gene of *S. epidermidis*.

As *S. epidermidis* biofilms exposed to NO showed higher lactate accumulation, we investigated whether this increase could be related to the induced expression of *ldh* gene encoding the l-lactate dehydrogenase enzyme (**Figure 1**). l-Ldh has been linked to drug resistance and is considered a possible target of regulatory control in many bacterial species, including in coagulase-negative staphylococci (Richardson et al., 2008; Toyoda and Inui, 2021; Ma et al., 2022; Yuan et al., 2022). For this purpose, biofilms of strains 1457 and RP62A, treated with NO or left untreated and grown for 24 h, were used to extract total RNA and evaluate *ldh* expression by RT-qPCR. Gene expression analysis of *ldh* in untreated RP62A and 1457 biofilm cells indicated a modest twofold higher expression in RP62A relative to 1457 cells (**Figure 5A**). However, a substantial approximately 50-fold higher expression of *ldh* in RP62A relative to 1457 was determined in NO-exposed biofilms (**Figure 5A**). Moreover, while in 1457 biofilm cells NO caused no significant variation in *ldh* expression, RP62A biofilms exposed to NO expressed *ldh* approximately 120-fold more than in untreated cells (**Figure 5B**). Importantly, the biofilm amounts and viabilities of 1457 and RP62A strains grown for 24 h with NO did not show significant differences between them (**Figure 2**).

Altogether, our results show that for biofilms at the same growth stage (after ~ 24 h), NO only induces the expression of *ldh* in strain RP62A, indicating the occurrence of different regulatory mechanisms regarding the production of lactate between RP62A and 1457.

### 2.3 Early biofilm formation of *S. epidermidis* is impaired by inhibition of lactate dehydrogenase

The role of lactate dehydrogenase in NO resistance of *S. epidermidis* biofilms was further confirmed in assays performed in the presence of oxamate, which is a pyruvate analog that inhibits the conversion of pyruvate to lactate (Altinoz and Ozpinar, 2022). For this purpose, biofilms were exposed to NO, as described above, in the presence of oxamate, and supernatants were analyzed by ^1^H-NMR to determine substrate consumption and metabolic end-products. Additionally, biofilm mass was measured by the crystal violet assay.

The biofilm mass of strain 1457 decreased in the presence of oxamate by approximately 34% (**Figure 2A**). Exposure to NO further lowered the biofilm mass formed at 24 h (**Figure 2A**). Oxamate also caused a reduction in viability of the biofilm-encased cells of strain 1457 by approximately 13-fold, which was further accentuated when the cells were under NO stress, resulting in a viability that was approximately 26-fold lower (**Figure 2B**). However, at 48 h, the pattern observed in strain 1457 changed, as oxamate addition to NO-treated biofilms caused a small increase in biofilm mass and a significant increase (approximately five times) in the viability of the encased cells (**Figure 2**).

Regarding RP62A cells, oxamate decreased the amount of biofilm mass formed by approximately 40%–46% regardless of the presence of NO and the age of the biofilm (**Figure 2A**). However, at 48 h, the effect of NO became noticeable, as the biofilm mass of cells treated with oxamate was greater when NO was present (**Figure 2A**), suggesting that strain RP62A undergoes modifications in the quantity of the biofilm matrix. In terms of viability, RP62A cells treated with oxamate and NO showed lower colony-forming unit (CFU) counts compared to cells not exposed to NO after 24 h, but this effect was not observed in 48-h biofilms (**Figure 2B**).

Metabolically, biofilms of strain 1457 grown for 24 h in the presence of oxamate denoted a substantial decrease of approximately 44% in consumed glucose and lactate levels (from 40 ± 2 to 8 ± 0 mM) and yields (61% less), relative to untreated biofilms (**Figures 3A, C**; **Table 1**). A significant shift toward acetate formation was also observed (**Figure 3E**, right panel; **Table 1**). Lactate and acetate accounted for approximately 31% and 22% of the glucose consumed, respectively (**Figures 3A, C, E** right panel; **Table 1**; **Figure 1**). In the RP62A strain, and unlike the 1457 strain, oxamate did not alter glucose consumption, and the decrease in lactate level (from 36 ± 0 to 24 ± 1 mM) and yield (1.5-fold lower) was not so drastic. Concerning acetate, the compound reached a concentration of 9 ± 1 mM, which was twofold higher compared to the control (**Figures 3B, D, F**; **Table 1**). Lactate and acetate accounted for approximately 46% and 17% of the glucose consumed, respectively (**Figures 3B, D, F**, right panel; **Table 1**). These results suggest a different regulatory mechanism for the production of lactate and recycling of NADH back to NAD^+^ (**Figure 1**) that allows strain RP62A to continue glycolysis at a higher extent, which does not occur in strain 1457. The effect of oxamate on lactate production of strains 1457 and RP62A is reflected in 15% lower ATP yields, relative to unexposed biofilms, despite the observed increase in acetate/ATP production (**Table 1**).

In strain 1457, oxamate/NO-treated biofilms, grown for 24 h, exhibited a significant decrease of approximately 54% in consumed glucose relative to NO-only-treated biofilms that was approximately 10% higher than the decrease caused by oxamate relative to control (**Table 1**; **Figure 3C**). Also, oxamate lowered substantially the lactate yield (by approximately 44%) and increased by approximately fourfold the acetate yields in oxamate + NO versus NO biofilms (**Table 1**; **Figures 3A, C, E**). However, unlike in oxamate-only-treated biofilms, in oxamate/NO-treated biofilms, lactate and acetate accounted for approximately 53% and 12% of the glucose consumed, respectively (**Table 1**), indicating that NO causes significant deviation of glucose to lactate production.

In biofilms of strain RP62A, no significant effect on glucose consumption was observed upon oxamate addition, but the lactate yield was 22% lower, denoting the negative effect of oxamate on lactate production (oxamate + NO versus NO in **Figures 3B, D**; **Table 1**). Nevertheless, this effect was much less pronounced (35% lower) than that observed in 1457 biofilms (**Figures 3A, B**; **Table 1**). For acetate, the trend was the opposite, aligning with the stimulating effect of oxamate on acetate production (**Figure 3F**; **Table 1**).

The addition of oxamate to NO-exposed biofilms lowered the ATP yields of both 1457 and RP62A biofilms by 23% and 13%, respectively, indicating energy production impairment caused by oxamate (**Table 1**).

In biofilms grown for 48 h, similar results were obtained when oxamate was added to NO-treated cells. The exception was the oxamate/NO-treated biofilms of strain 1457, as glucose consumption was not complete and ~ 40% lower than that observed in biofilms exposed to oxamate or NO alone (**Figure 3C**; **Table 1**). Moreover, the lactate amounts did not increase substantially from 24 to 48 h, contrary to oxamate-only-exposed biofilms, suggesting a reverse effect of NO on lactate production by Ldh in 1457 biofilms in the presence of oxamate (**Figure 3A**; **Table 1**).

Hence, we concluded that inhibition of lactate production of lactate dehydrogenase by the competitive inhibitor oxamate leads to significant impairment in biofilm mass formation of early biofilms (24 h) of *S. epidermidis*, treated and untreated with NO.

### 2.4 Confocal microscopy analysis of *S. epidermidis* biofilms reveals that nitric oxide interferes with matrix polymers production

Biofilm mass and end-products amounts are modified by NO (**Figures 2**, **3**). These results led us to surmise that the levels of metabolite intermediates linked to glycolysis, the amino sugar pathway, and the synthesis of PIA, which is known to be a substantial part of the matrix of several *S. epidermidis* biofilms (**Figure 1**), are also affected by NO. Therefore, we used RT-qPCR to determine in biofilms the expression of genes of the amino sugar pathway (*glmM* and *glmU*) involved in the production of PIA precursors and those involved directly in PIA production (e.g., *icaA*). Additionally, we investigated the NO effect on staphylococcal biofilm matrix by combining confocal laser scanning microscopy (CLSM) and staining of the major biofilm matrix components, namely, exopolysaccharides/PIA and proteins, with the WGA and Sypro Ruby fluorophores, respectively. These assays allow the direct visualization of the localization of main matrix components and the determination of their amounts in biofilm aggregates.

#### 2.4.1 Biofilm matrix polymers

**Figure 6:**
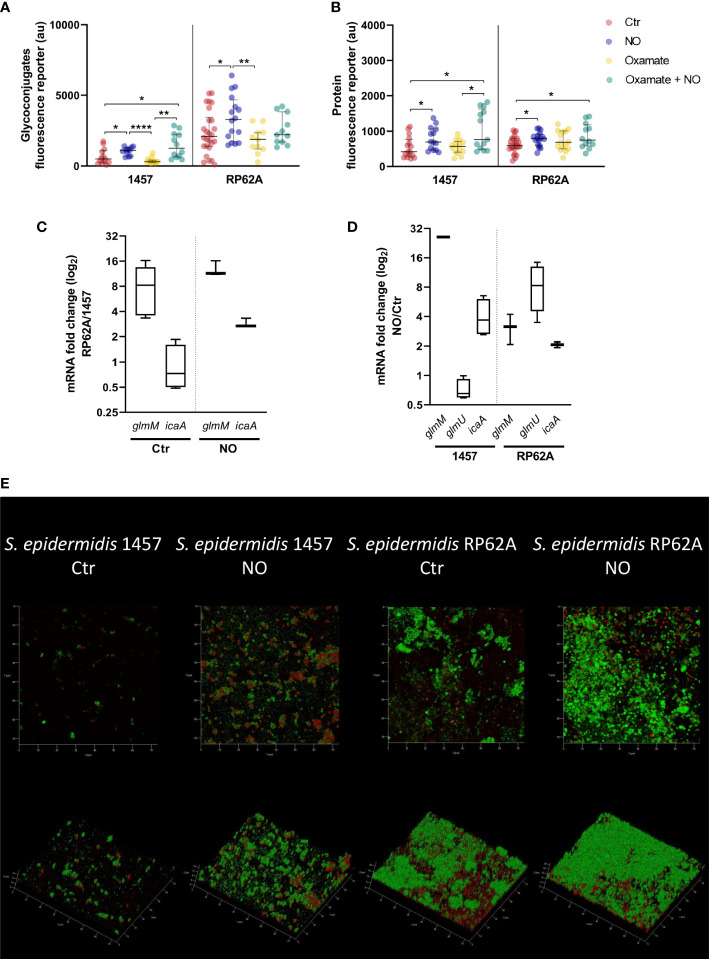
Amount of matrix polymers and NO-induced expression changes in biofilms of *Staphylococcus epidermidis* exposed to NO. Quantification by confocal microscopy of the amount of exopolysaccharides **(A)** and proteins **(B)** in the matrix of 24-h biofilms of *S. epidermidis* strains 1457 and RP62A in the presence (blue dots) and absence (Ctr, red dots) of 1 mM of NO and 5 mM of oxamate (yellow dots) and oxamate+NO (green dots). Log-2-fold changes in gene expression of **(C)** *glmM* and *icaA* from 24-h biofilms of strain RP62A as compared to strain 1457, in the absence and presence of NO, and **(D)** *glmM*, *glmU*, and *icaA* of 1457 and RP62A biofilms with NO as compared to control without NO addition. **(E)** Confocal images, depicted as Z and orthogonal projections, representative of the *S. epidermidis* 1457 and RP62A biofilms exposed or unexposed (control) to 1 mM of NO, with matrix proteins and polysaccharides in red and green, respectively. Each image shows a 75 × 75 µm section of the biofilm with varying heights. Each component was detected by staining with appropriate fluorophores. Scattered symbols represent individual measurements (*n* ≥ 15 for protein and exopolysaccharides), and horizontal lines indicate median values and interquartile range. All comparisons were performed using t-tests with Welch’s correction, when appropriate. Asterisks represent statistically significant data relative to control (****, *p* ≤ 0.0001; **, *p* ≤ 0.01; *, *p* ≤ 0.05).

Biofilms of RP62A contain three times more exopolysaccharides in the matrix per biofilm area than those of the 1457 strain (*p*-value < 0.0001, Welch’s t-test) (**Figures 6A, E**). This difference might be a direct consequence of the higher *glmM* transcript levels in RP62A (~8x higher), as *icaA* expression and glucose consumption were identical in both strains (**Figures 1**, **3C, D**, **6C**). Regarding the protein amounts, no significant difference was observed between the strains (**Figure 6B**).

Interestingly, both proteins and exopolysaccharides/PIA amounts were significantly higher in biofilm matrices of the clinical strains exposed to NO (**Figures 6A, B, E**), as were the expression of genes of the amino sugar pathway (*glmM* and *glmU* in RP62A and *glmM* in 1457) and synthesis of PIA (*icaA*) (**Figures 1**, **6D**). To understand if this effect was dependent on cells performing increased lactate production in the presence of NO, we exposed biofilms to oxamate. Interestingly, although on average it was not affected by oxamate, in strain 1457, the amounts of exopolysaccharides and proteins in the matrix of oxamate/NO-exposed biofilms were dispersed toward higher values upon oxamate exposure (**Figures 6A, B**, **S6**). This dispersion led to the formation of two visible populations, one with amounts similar to those obtained for oxamate-untreated/NO-treated biofilms and another with higher amounts (**Figures 6A, B**, **S6**). These results suggest that the production of lactate, PIA, and matrix proteins are independent NO-stimulated processes.

#### 2.4.2 Biofilm structural matrix phenotype

**Figure 7:**
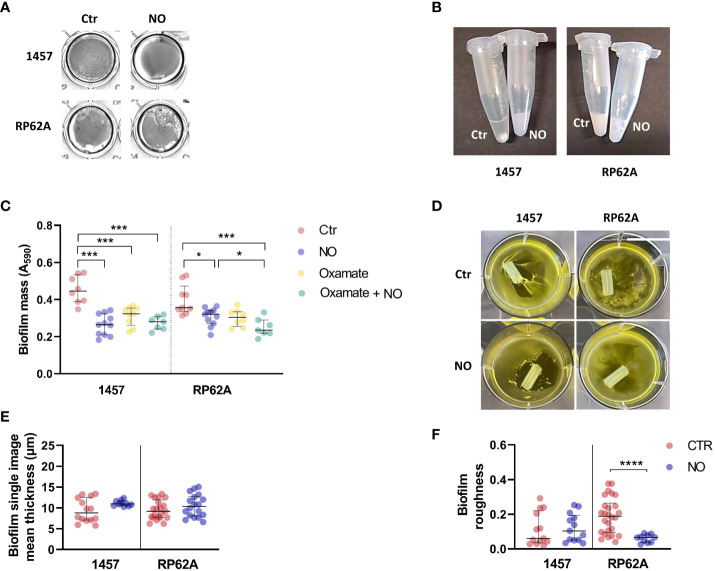
Structural properties and phenotype of the biofilms of *Staphylococcus epidermidis* 1457 and RP62A strains exposed to NO. 1457 and RP62A biofilms stained with crystal violet **(A)** and resuspended in PBS (1×) inside Eppendorf tubes **(B)** with or without (Ctr) the addition of 1 mM of NO. Both strains were grown in contact with silicon catheter-mimicking tubes, in high-glucose DMEM/FBS medium for 24 h, and in the absence (Ctr, red dots) or presence of 1 mM of NO (blue dots) and 5 mM of oxamate (yellow dots) and oxamate+NO (green dots). The amount of biofilm attached to the tube was measured by crystal violet staining **(C)**. At 24 h of growth in the same conditions, visible alterations in the biofilm were present in the absence (Ctr) *versus* presence of NO **(D)**. Each figure shows a representative experiment of an *n* ≥ 7. Single image means thickness (µM) **(E)** and roughness **(F)** of the biofilm matrices of individual confocal images of 1457 and RP62A biofilms unexposed (Ctr, red dots) and exposed to NO (blue dots). Extracellular proteins and polysaccharides were visualized by staining with appropriate fluorophores. Scattered symbols represent individual measurements (*n* ≥ 15), and horizontal lines indicate median values and interquartile range. All comparisons were performed using Welch’s t-tests. Asterisks represent statistically significant data relative to control (****, *p* ≤ 0.0001; ***, *p* ≤ 0.001; *, *p* ≤ 0.05). PBS, phosphate-buffered saline; DMEM, Dulbecco’s modified Eagle medium; FBS, fetal bovine serum.

The alterations caused by NO on *S. epidermidis* biofilm matrices were visible by macroscopic examination of the biofilms and their manipulation. In the absence of NO, 1457 biofilms formed visible cell aggregates at the bottom of cell culture plate wells (**Figure 7A**), which were difficult to be resuspended in phosphate-buffered saline (PBS) (**Figure 7B**) but relatively loose from the wells. Strain 1457 exposed to NO showed an even, smooth, and unstructured appearance throughout the entire biofilm (**Figure 7A**), which was preserved in the presence of oxamate (**Figure S7**), reinforcing that the biofilm phenotype is independent of the lactate dehydrogenase activity. This phenotype, together with the lower biofilm amounts in the presence of NO and oxamate, was maintained when biofilms were grown in contact with silicone catheter pieces (**Figures 7C, D**). The biofilms also became more attached to the wells in the presence of NO. This striking difference between unexposed and NO-treated biofilms might be explained in part by the increase of the positively charged PIA exopolysaccharide in the matrix of 1457 NO-exposed biofilms (**Figures 6A, E**), which is attracted to the negatively charged surfaces of polystyrene plates and silicone, thus causing higher biofilm adherence.

Parameters such as thickness and roughness of the biofilm matrix were measured by confocal microscopy at several points of the biofilm surface, which is highly heterogeneous (López-Guerra et al., 2019; Beckwith et al., 2020). The data thus extracted showed that the thickness of 1457 biofilm matrices, although not significantly affected by NO in mean terms, became more homogenous in the entire biofilm, forming one sole population with higher thickness values (**Figure 7E**). This result agrees with the more even and smooth surface of the biofilms (**Figures 7A**).

Strain RP62A grown in the absence of NO formed bumpy biofilms at the bottom of the cell culture plate wells and in contact with silicone catheter (**Figures 7A, D**), which were easy to be resuspended in PBS (**Figure 7B**) but difficult to detach, probably due to their higher content in positively charged PIA (**Figures 6A, E**). In the presence of NO, the macroscopic appearance of the RP62A biofilms was in terms of texture similar to that of unexposed cells. Nevertheless, more cavities and fragmented biofilm were visible (**Figure 7A**). Microscopically, the RP62A biofilm matrices were significantly rougher than the matrices of 1457 (*p*-value 0.0292, Welch’s t-test) (**Figure 7F**), but in the presence of NO, the roughness of RP62A biofilm matrices decreased by threefold (*p*-value 0.0004) and 1.6-fold (*p*-value 0.0182) relative to control and 1457, respectively (**Figure 7F**).

Altogether, our data show that nitric oxide increases PIA production and the production of exopolysaccharides and proteins in the biofilm matrices of *S. epidermidis* clinical strains, causing alterations in the biofilm’s architecture.

### 2.5 Biofilms of *S. epidermidis* performing nitrite respiration show similar metabolism as those exposed to NO stress

**Figure 8:**
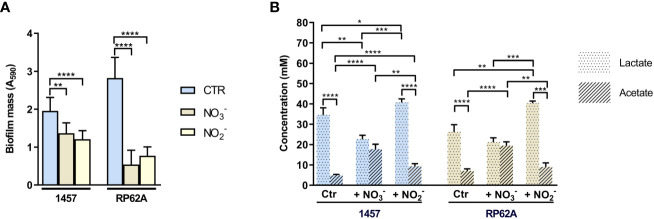
Effect of nitrate and nitrite on mass and end-products profile of biofilms of *Staphylococcus epidermidis* 1457 and RP62A. **(A)** Amounts of biofilm in 1457 and RP62A strains after 48-h growth in the absence (Ctr, blue bars) and presence of nitrate (NO_3_^−^, brown bars) and nitrite (NO_2_^−^, yellow bars). Biofilm mass was assessed using the crystal violet assay, measured by absorbance at 590 nm. Error bars represent mean ± SD (*n* = 6 for Ctr and *n* = 3 for the +NO_3_^−^ and +NO_2_^−^ conditions). **(B)** Major extracellular metabolites accumulated by biofilms of *S. epidermidis* 1457 and RP62A strains grown for 48 h in high-glucose DMEM/FBS, in the absence (Ctr) and presence of 40 mM nitrate (NO_3_^−^) and 5 mM nitrite (NO_2_^−^). Symbols: pointed bars, lactate; hatched bars, acetate. Comparisons were performed using Welch’s t-tests. Asterisks represent statistically significant data (****, *p* ≤ 0.0001; ***, *p* ≤ 0.001; **, *p* ≤ 0.01; *, *p* ≤ 0.05).

In hypoxic (low oxygen) microenvironments, *S. epidermidis* can grow by fermentation or alternatively by nitrate/nitrite respiration when nitrate/nitrite is available. In the absence of oxygen or under low oxygen tensions, the staphylococcal oxygen-reducing terminal oxidases of the aerobic respiratory chain are replaced by nitrate and nitrite reductases (Nar and Nir), which are involved in the anaerobic dissimilatory nitrate and nitrite reduction (**Figure 1**). During anaerobic nitrate and nitrite respiration, NO is formed from the activity of nitrite reductase on nitrite (**Figure 1**). Since we observed that exogenous NO led to an increase in lactate production (**Figure 3**; **Table 1**), we wondered what would be the effect of NO produced endogenously from nitrite reduction. We observed that during nitrate and nitrite respiration under hypoxic conditions, the biofilm formed by strains 1457 and RP62A decreased by 1.5- and 5-fold relative to the control (i.e., biofilms grown without nitrate or nitrite), respectively (**Figure 8A**). We determined the metabolism of 1457 and RP62A biofilms grown for 48 h under static conditions (control, Ctr) and observed that they consumed all the glucose available in the medium (26 mM) and excreted lactate (approximately 26–36 mM) as the major end-product (**Figure 8B**), which is consistent with a fermentative metabolism for carbon/energy production. Together with lactate, acetate was ubiquitously excreted, but in smaller amounts (approximately 4–7 mM) (**Figure 8B**), and minor quantities of ethanol, 2,3-butanediol, formate, and succinate were detected (data not shown).

When nitrate was used as the terminal electron acceptor, a significantly higher shift to production of acetate occurred (to approximately 20 mM) concomitantly with the decrease in lactate accumulation that reached a concentration of only approximately 20 mM (**Figure 8B**). Despite the synthesis of ATP that accompanies acetate production and the formation of electrochemical gradients generated from the electron transfer systems during anaerobic respiration (**Figure 1**), which make nitrate respiration energetically more favorable than fermentation, the biofilm masses of the strains were lower than those formed under lactic fermentation (**Figure 8A**).

Strain 1457 had a different response when nitrite was used as an alternative electron acceptor, as the increase in acetate formation was modest relative to the control (**Figure 8B**). However, there was a significant increase in lactate excretion in both strains, compared to the control and nitrate-respiring biofilms (**Figure 8B**). These results indicate that nitrite is not an efficient electron acceptor of the respiratory chain as is nitrate; thus, unlike nitrate reduction, nitrite reduction was not able to efficiently couple to NADH oxidation at the level of the respiratory chain to generate a proton motive force and restore redox balance (**Figure 1**). Instead, nitrite restores the redox balance at the level of lactic fermentation, an effect that is similar to that observed under fermentative conditions in the presence of NO. In this way, nitrite reduction deviates glucose to the production of lactate, which is detrimental to the production of acetate, a step where ATP, instead of NAD^+^, is produced.

### 2.6 In *S. epidermidis* biofilms lactate production is also stimulated by NO-proficient macrophages

**Figure 9:**
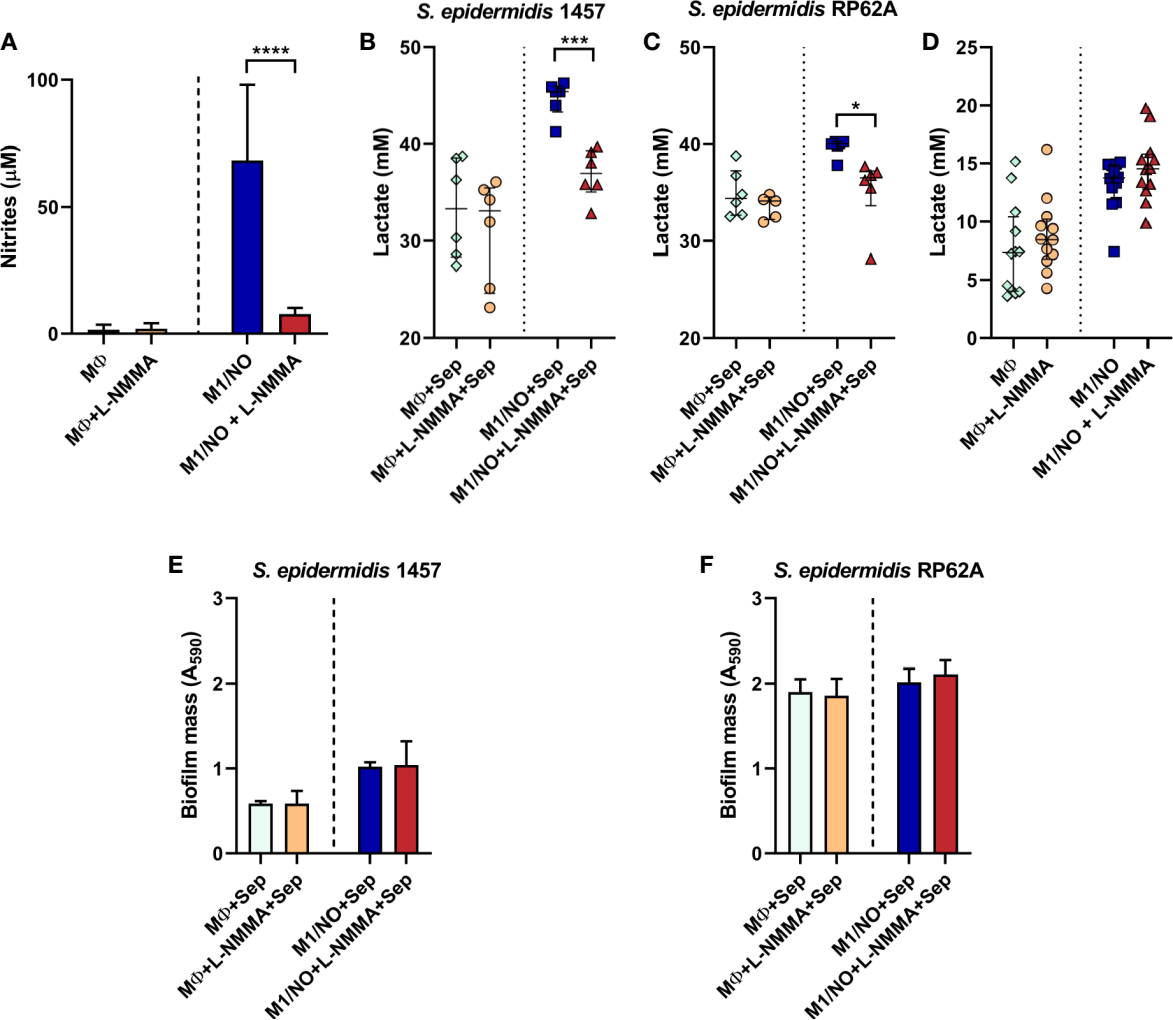
Biofilm mass, extracellular nitrites, and lactate accumulated in co-cultures of *Staphylococcus epidermidis* biofilms with NO-proficient macrophages. **(A)** Evaluation of the extracellular nitrite accumulation in the supernatants of l-NMMA-untreated (light blue and dark blue bars), l-NMMA-treated (orange and red bars), M1-activated (NO, dark blue and red bars), and non-activated (light blue and orange bars) macrophages (MØ) before infection with *S. epidermidis*. **(B, C)** Lactate accumulation in the supernatants of l-NMMA-untreated (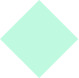, 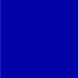) and l-NMMA-treated (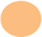, 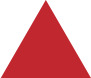), M1-activated (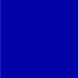, 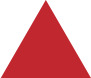), and non-activated (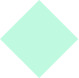, 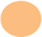) macrophages (MØ) co-cultivated with *S. epidermidis* strains 1457 **(B)** and RP62A **(C)** in inserts/transwells for 24 h in high-glucose DMEM/FBS medium. **(D)** Lactate accumulation in the supernatants of l-NMMA-untreated (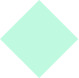, 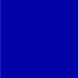) and l-NMMA-treated (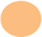, 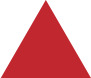), M1-activated (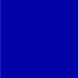, 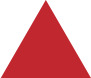), and non-activated (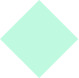, 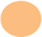) macrophages (MØ) grown for 24 h. Nitrites were determined by the Griess method, and lactate concentrations were determined by ^1^H-NMR. **(E, F)** Biofilm formed by *S. epidermidis* 1457 **(E)** and RP62A **(F)** grown for 24 h in high-glucose DMEM/FBS medium in the presence of M1-activated (dark blue and red bars) and MØ non-activated (light blue and orange bars) macrophages that were untreated (light and dark blue bars) or l-NMMA-treated (orange and red bars). Biofilm mass was determined using the crystal violet assay measured by absorbance at 590 nm. **(A, E, F)** Error bars represent mean ± SD (*n* = 6 for biofilm mass and *n* = 18 for nitrites). **(B–D)** Scattered symbols represent individual measurements, and horizontal lines indicate median values and interquartile range (*n* = 6). Comparison was performed using Welch’s t-tests. Asterisks represent statistically significant data relative to control (****, *p* ≤ 0.0001; ***, *p* ≤ 0.001; *, *p* ≤ 0.05). DMEM, Dulbecco’s modified Eagle medium; FBS, fetal bovine serum.

The lactate production by *S. epidermidis* strains while exposed to murine macrophages J774A.1 and forming biofilms was analyzed. Since macrophages carry out lactic fermentation, lactate was also measured in macrophages cultured in the absence of bacteria to serve as the control. Production of NO by M1 macrophages and the inhibitory effect of iNOS were confirmed by nitrite determination in the macrophages’ supernatants (**Figure 9A**). As expected, macrophages not stimulated or inhibited in NO production, designated as MØ or MØ+l-NMMA, respectively, accumulate negligible amounts of nitrite (approximately 1.5–1.9 mM), while activated macrophages (aka M1/NO) accumulated 35–45 times more nitrite (**Figure 9A**). Inhibition of activated macrophages by l-NMMA (M1/NO+l-NMMA) resulted in non-significant values of nitrite formed, reflecting the non-production of NO (**Figure 9A**).

Analysis of the macrophage/bacteria co-culture showed that, although glucose consumption did not change, significantly more lactate is accumulated when the biofilms of strains 1457 and RP62A are formed (at 24 h) in the presence of activated macrophages (M1/NO) than when in contact with iNOS-inhibited macrophages (M1/NO+l-NMMA) (**Figures 9B, C**, **S8A, B**). Moreover, the levels of acetate and formate formed were negligible (data not shown).

Importantly, the increase in lactate in co-cultures of bacteria/activated macrophages M1 is directly related to NO production since there is no variation in lactate amount formed in co-cultures of bacteria/non-activated macrophages incubated with the l-NMMA inhibitor (**Figures 9B, C**). We concluded that bacteria are responsible for increasing lactate production, as observed in biofilms from co-cultures of bacteria/NO-producing macrophages (**Figures 9B, C**) in which l-NMMA did not affect the amount of lactate formed or glucose consumed in single cultures of NO-producing macrophages (**Figures 9D**, **S8C**).

Our data indicate that in *S. epidermidis* biofilms, the NO released from macrophages promotes a pronounced shift to lactic fermentation. Interestingly, in both staphylococcal strains, the amount of biofilm formed was not affected by the NO-producing macrophages (**Figures 9E, F**), suggesting that the increased lactate production could be one of the factors involved in the NO resistance of bacteria.

## 3 Discussion

Although the ability of *S. epidermidis* to form biofilms is what underpins its pathogenic trait, the mechanisms that allow this microorganism to efficiently transit from a planktonic lifestyle to a biofilm under the hostile environment generated by the host’s produced toxic chemicals, such as NO, remain incompletely defined. In this work, we characterized the phenotypic and metabolic response to NO stress of two PIA-biofilm-forming model strains of *S. epidermidis*, namely, 1457 and RP62A, while growing in biofilms in a serum- and glucose-containing medium that sustains bacterial growth and biofilm formation and is physiologically relevant for the expression of iNOS in M1 macrophages.

We observed that externally added NO decreases the amount and alters the macroscopic features of biofilms formed from planktonic cells of strains 1457 and RP62A cultivated in static *in vitro* models of biofilm. In other microorganisms, such as *S. aureus*, *P. aeruginosa*, and *Acinetobacter baumannii*, micromolar concentrations of NO also prevented biofilm formation (Sulemankhil et al., 2012; Ren et al., 2016; Namivandi-Zangeneh et al., 2018; Li et al., 2022).

Although exposure to sub-lethal NO concentration leads to a lower number of viable cells in the *S. epidermidis* biofilm, and consequently reduced mass, full growth impairment was not observed, and the metabolism underlying the growth of NO-resistant biofilm-forming cells proved to be different from that of unexposed biofilms.

NO resistance of *S. epidermidis* biofilms of strains 1457 and RP62A grown under fermentative conditions is shown here to be achieved through a significant rise in Ldh-derived lactate production from glucose metabolism. The concentration of glucose in our medium is approximately 26 mM. This concentration is substantially higher than the homeostatic concentration of glucose in the blood (3–5 mM), which is the *in vivo* context of most *S. epidermidis* biofilm infections. However, it might not influence the metabolism of the bacterium, as glucose in the blood is constantly available. Indeed, we show that the rise in lactate is observed not only under excess glucose (26 mM) but also in glucose-limiting conditions (in TSB).

In *S. aureus*, an increase in lactate production was also observed in aerobically grown planktonic cells exposed to nitrosative stress (Richardson et al., 2008). Moreover, Ldh and several glycolytic enzymes were reported to be active in cells of biofilm grown statically (Martínez-García et al., 2021). However, planktonic cells of *S. epidermidis* commensal strains 6293 and 6903 grown in the presence of NO do not replicate or have lactate dehydrogenase activity (Richardson et al., 2008).

We show that externally added nitrite and nitrate impair biofilm mass formation in both strains. In *S. epidermidis*, the nitrate and nitrite reductases encoded in the genomes of strains 1457 and RP62A would allow, under anaerobic conditions, the conversion of nitrate into nitrite and nitrite into NO, respectively (Fuchs et al., 2007; Niemann et al., 2014; Durand and Guillier, 2021). We observed that nitrite reduction is mainly coupled to NAD^+^ regeneration at the lactate dehydrogenase level, and like NO, nitrite increases Ldh-lactate production by both strains. Thus, we propose that endogenous NO, derived from nitrite, is responsible for the higher lactate amount present in nitrite-grown biofilms. Consistent with these results, nitrite-mediated inhibition of *S. aureus* biofilm formation was previously reported to be abrogated by the addition of NO scavengers (Schlag et al., 2007).

The expression of the l-lactate dehydrogenase *ldh* gene, localized in the *ldh–alsS–budA* operon in strains 1457 and RP62A, was highly induced in NO biofilms of strain RP62A but, remarkably, not affected in strain 1457, despite that both strains exhibited a rise in lactate production under NO stress. A reasonable explanation for this difference is difficult to put forward. However, *S. epidermidis* strains 1457 and RP62A exhibit three allelic differences in the gene found upstream of the *ldh–alsS–budA* operon, coding a LysR-type regulator, which might be responsible for the different transcriptional regulatory activities. The *S. epidermidis* LysR-type regulator is highly homologous to the *S. aureus* CidR regulator, which was previously shown in *S. aureus* to respond to NO and to control the expression of the *alsS*/*budA* genes (Carvalho et al., 2017; Sadykov et al., 2019). The higher lactate levels found in NO-stressed biofilms of the 1457 strain may result from post-translational regulation, as the lack of correlation between the levels of gene transcription and metabolic behaviors derived from allosteric regulation of metabolism has been reported (Carvalho et al., 2011). Another explanation for the observed increase in lactate production in 1457-forming biofilms exposed to NO could be that lactate derives from d-lactate dehydrogenase rather than l-lactate dehydrogenase, whose activities lead to the formation of d-lactate and l-lactate isomers, respectively, that cannot be distinguished in the ^1^H-NMR spectra, as they resonate in similar spectral regions under the NMR-acquiring conditions. However, to our knowledge, in contrast with L-*ldh*, transcriptional regulatory mechanisms of D-*ldh* genes have never been reported for Gram-positive bacteria (Feldman-Salit et al., 2013; Toyoda and Inui, 2021; Ma et al., 2022).

We identified a NO-induced decrease in the extracellular accumulation of acetate and formate. This trend is probably a consequence of the deleterious effect of NO on the activities of enzymes acting downstream pyruvate, especially the anaerobic pyruvate formate-lyase (PFL), which limits the acetyl-coA required for acetate production by acetate kinase (AckA) (Richardson et al., 2008; Troitzsch et al., 2021), and may play a role in directing energy from pyruvate to lactate production. Nevertheless, direct NO-mediated regulation on *ackA* should not be excluded. Recently, an analysis of invasive *S. epidermidis* showed that subpopulations characterized by infection-supporting phenotypes (e.g., increased biofilm formation and antibiotic resistance) carry mutations in *ackA*, probably to divert carbon flux from acetyl-coA to alternative metabolic pathways, such as the TCA cycle or glycolysis (Both et al., 2021).

Succinate is a product excreted from the activity of the reverse TCA cycle, which operates under anaerobic conditions in several microorganisms (Fuchs et al., 2007). We found only a minor amount of succinate accumulated in strains 1457 and RP62A biofilms, suggesting a minor activity of the reverse TCA cycle. In agreement, the proteins involved in the reductive reactions of the reverse TCA cycle were not detected in a proteomic analysis of *S. epidermidis* biofilms (Martínez-García et al., 2021). The addition of fluorocitrate, an inhibitor of the oxidative TCA cycle aconitase A (AcnA) enzyme, to biofilm-growing cells of *S. epidermidis* did not affect biofilm mass production (data not shown), which indicates that AcnA and consequently the oxidative TCA cycle do not seem to be important for biofilm formation. Moreover, the *S. epidermidis* oxidative TCA cycle activity and aerobic respiratory chain enzymes are repressed under low oxygen tensions (Vuong et al., 2005; Uribe-Alvarez et al., 2018; Pedroza-Davila et al., 2020).

The exposure to NO of strains 1457 and RP62A forming biofilms resulted in enhanced expression of genes of the amino sugar pathway (such as *glmM* and *glmU*), which link glycolysis to PIA production, and *icaA*, involved in PIA exopolysaccharide biosynthesis. By using CLSM, we demonstrated that NO augments not only exopolysaccharide amounts in the biofilm matrix of those strains but also the protein amounts. Biofilm formation is a highly dynamic and energetically costing process that, in addition to PIA, also involves the production and secretion of proteins (Nguyen et al., 2020; Schilcher and Horswill, 2020; Calvo et al., 2022). In *S. epidermidis*, enhanced PIA production has been associated with decreased TCA cycle activity caused by several stresses (e.g., low iron, high salt, ethanol, and heat), together with redirection of carbon to glycolysis (Vuong et al., 2005; Sadykov et al., 2011; Zhang et al., 2011; Martínez-García et al., 2021). These studies were conducted in aerobically grown planktonic cells (Vuong et al., 2005; Sadykov et al., 2011; Zhang et al., 2011) where the TCA cycle has significant activity. However, the biofilm promotes a hypoxic environment, which implies that the increase in PIA production caused by NO certainly does not derive from the decrease in TCA cycle activity.

In strain 1457, biofilms formed at 24 h and exposed to NO had several growth and energy parameters (e.g., biofilm formation and viability, glucose consumption, lactate production, and ATP yields) decreased by inhibition of Ldh activity by oxamate and even significantly more decreased than when oxamate alone was applied to biofilms. However, this multifactorial impairment was not observed for strain RP62A grown under the same conditions, probably due to the different regulatory mechanisms of lactate production, which allow the strain to meet the lactate/NAD^+^ requirement by increasing Ldh expression. Interestingly, in what regards biofilm amounts, the opposite is observed when oxamate and NO-exposed biofilms are formed in catheter-simulating silicon tubes, which suggests that the metabolic events linked to biofilm development are modulated by the adherent surface.

In the biofilm-forming *S. aureus*, oxamate did not inhibit biofilm growth and lactate production (Heim et al., 2020). However, *S. aureus* possesses *ldh1* gene whose expression is triggered by redox imbalance and leads to increased l-lactate/NAD^+^ production (Richardson et al., 2008; Pagels et al., 2010; Heim et al., 2020). Our results suggest that in NO-exposed *S. epidermidis* biofilms, the increase in lactate/NAD^+^, accompanied by a decrease in acetate/ATP excretion, is an adaptive metabolic response that allows NAD^+^ recycling for glycolysis and redox equilibration. Moreover, it provides significant levels of matrix precursors and ATP yields to produce large amounts of protective exopolymer layers, including PIA. It should be noted that PIA and peptidoglycan, which are essential for cell division, need the same precursors of the amino sugar pathway for their synthesis (Zhu et al., 2009; Schoenfelder et al., 2019). Indeed, it is reported that staphylococci prioritize biofilm matrix/PIA production over cell division under low oxygen concentrations (Pedroza-Davila et al, 2020). Additionally, increased production of matrix polymers, such as PIA, and modifications in the architecture of biofilms of bacteria have a protective role against antibiotics and other stresses (Oliveira et al., 2022; Severn and Horswill, 2023).

In this study, we revealed the metabolic strategies that *S. epidermidis* forming biofilms use to survive NO stress. Additionally, bacterial NO resistance is also dependent on efficient detoxification/repair systems, with flavohemoglobin (Hmp) being a key NO detoxifier as shown for several planktonically grown bacteria (Poole, 2020; Carvalho et al., 2021; Pidwill et al., 2021). We also found that Hmp is highly induced in strains 1457 and RP62A growing in biofilms exposed to NO (data not shown). We previously reported a macrophage biofilm-forming bacteria co-culture model that shows the contribution of *S. aureus* Hmp to the survival of a biofilm at an early stage of formation when under nitrosative stress (Carvalho et al., 2021). In this work, using the same co-culture model, we showed that biofilms of strains 1457 and RP62A formed in contact with NO-proficient macrophages have significantly increased lactate production so as not to compromise the formation of biofilm mass.

## 4 Conclusion

**Figure 10:**
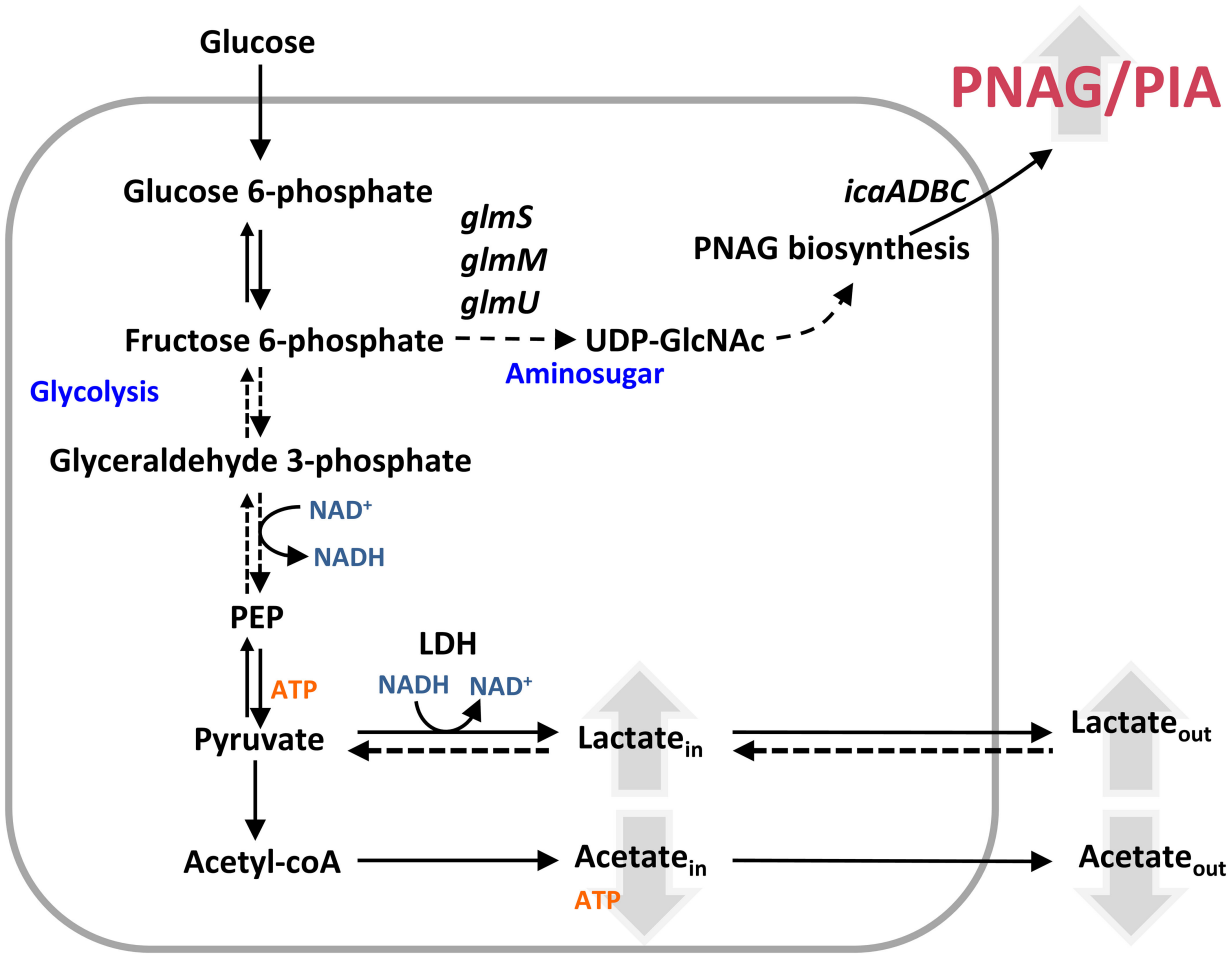
Model of nitrosative stress effect on *Staphylococcus epidermidis* biofilms. NO increases the amount of excreted lactate, whose production is coupled with NADH oxidation to NAD^+^, and decreases the secretion of acetate. The available NAD^+^ allows glycolysis to continue and feeds fructose-6-phosphate necessary for PIA production. Effect of NO in molecules is represented by upward (increase) and downward (decrease) pointing arrows. *glmS*, glutamine fructose-6-phosphate transaminase; GlmM, phosphoglucosamine mutase; *glmU*, glucosamine 1-phosphate *N*-acetyltransferase; *icaA*, poly-beta-1,6-*N*-acetyl-d-glucosamine synthase; Ldh, lactate dehydrogenase; PEP, phosphoenolpyruvate; PIA, polysaccharide intercellular adhesion; PNAG, poly-β-1,6-*N*-acetyl-d-glucosamine; UDP-GlcNAc, UDP-*N*-acetylglucosamine.

The effect of NO on bacterial biofilms is for the first time presented here. We propose the following model of how *S. epidermidis* adapts to NO while growing in biofilms (**Figure 10**): NO decreases acetate excretion and stimulates lactate/NAD^+^ production. The boost of NAD^+^ from lactate production permits glycolysis to proceed, *via* consumption of NAD^+^ at the level of glyceraldehyde 3-phosphate dehydrogenase and regeneration of more glyceraldehyde phosphate in the first half of glycolysis, and feed fructose 6-phosphate for the amino sugar production. Amino sugar and PIA production are upregulated by NO. Nitric oxide decreases biofilm mass, but the matrix of the biofilms, which have a protective role against antimicrobials, presents more PIA and proteins, as well as altered architecture. We showed that stimulation of lactate production also occurs in *S. epidermidis* growing in biofilms in contact with NO-producing macrophages, unveiling that Ldh may have a protective role against NO *in vivo*.

Basic microbial physiology and metabolism and virulence are closely intertwined (Poquet et al., 2018; Gu et al., 2022; Yue et al., 2022; Srimahaeak et al., 2023). Our results enhance the understanding of how *S. epidermidis* biofilms resist NO and contribute to finding mechanisms to inhibit the fitness of biofilm formation *in vivo*. Oxamate, an isosteric form of pyruvate, is currently used in malignant cells, which are mostly shifted to anaerobic metabolism, to inhibit lactate dehydrogenase and thus the progression of cancer in animal models (Valvona and Fillmore, 2018; Qiao et al., 2021; Altinoz and Ozpinar, 2022). A clinical limitation of oxamate is its low penetrance through animal cell membranes (Altinoz and Ozpinar, 2022), which can be beneficial if oxamate is considered as an adjuvant of NO released from biomaterials or nanoparticles, that have been used to treat bacterial biofilm infections (Rong et al., 2019; Chiarelli et al., 2021; Chug and Brisbois, 2022).

## 5 Materials and methods

### 5.1 *S. epidermidis* strains, media, and growth conditions

Strains of *S. epidermidis* used in this study, listed in **Table S1**, were stored in 25% (v/v) glycerol (Merck, Darmstadt, Germany) at −80°C. Pre-inoculum cultures of *S. epidermidis* were routinely started at 1% (v/v), from −80°C stocks, in TSB (Difco, Franklin Lakes, NJ, USA) and grown to exponential phase (approximately 4–5 h) at 37°C and 150 rpm in Erlenmeyer flasks.

*S. epidermidis* biofilms were grown statically, at 37°C, in 2 ml of DMEM containing 4.5 g/L of glucose, 862 mg/L of l-alanylglutamine, 110 mg/L of sodium pyruvate, and 15 mg/L of phenol red (Gibco, Amarillo, TX, USA; ref. 31966021), supplemented with 10% heat-inactivated FBS (Gibco, ref. 10270106) on 24-well polystyrene tissue culture plates (Sarstedt, Nümbrecht, Germany). Where applicable, sodium nitrate and sodium nitrite were added at final concentrations of 40 and 5 mM, respectively. Alternatively, biofilms were grown on 96-well plates (Sarstedt) containing 200 µl of TSB. The media were inoculated with exponential cells of *S. epidermidis* to an OD_600_ of 0.05 before the filling of the wells (3–4 wells per strain and condition). Following 2 h of incubation at 37°C, the cells (approximately 10^7^ CFUs/ml) were left untreated or exposed to the indicated concentration of DETANONOate (ACROS Organics, Geel, Belgium) and oxamic acid sodium salt (Alfa Aesar, Ward Hill, MA, USA), freshly dissolved in 0.01 M of NaOH and H_2_O, respectively, and the plates were incubated longer for 22 or 46 h.

*S. epidermidis* planktonic cells were grown at 37°C and 150 rpm, in 20 ml of DMEM/FBS medium described above. The media were inoculated with exponential cells of *S. epidermidis* to an OD_600_ of 0.05, and following 2 h of incubation at 37°C and 150 rpm, the cells were left untreated or exposed to 1 mM of DETANONOate.

### 5.2 Determination of biofilm and free cell biomasses and viabilities

*S. epidermidis* biofilms were grown for 24 and 48 h in 24- and 96-flat well plates in DMEM plus FBS and TSB, respectively, as described above. After the incubation periods, the media covering the biofilms of each well were removed, and whenever relevant, OD_600_ measurements were performed to access free cell biomass. Subsequently, biofilms were heat-fixed at 65°C for at least 2 h and stained with crystal violet (CV), as follows: 2 ml (24-well plates) or 200 µl (96-well plates) of CV (1% (v/v), Merck) was added to wells followed by incubation at room temperature (RT), for 20 min. Biofilms were washed twice with 1 volume of PBS (1×), and images were acquired with an ImageScanner III LabScan 6.0 (600 dpi, GE Healthcare, Chicago, IL, USA). PowerPoint was used to enhance the visualization of the biofilms on the plates, by increasing the brightness by 40% and/or using the sharpen function at 50%. To elute the crystal violet, 1 volume of glacial acetic acid (33% (v/v), Carlo Erba, Milan, Italy) was then added. After 10-min incubation, the CV absorption was measured at 590 nm.

The viability of the biofilm-encased and biofilm-free cells was determined as follows. The medium covering the biofilms in each well was collected into microcentrifuge tubes and homogenized before serial dilutions and plating on Tryptic Soy Agar (TSA). Biofilms extracted from the 24- and 96-well plates were collected in microcentrifuge tubes containing 1 ml and 100 µl of PBS (1×), respectively. Biofilms were vortexed for 30 s, sonicated (Bioruptor^®^ Plus Sonication System) for 12 cycles (10 s ON, 30 s OFF, on high setting), vortexed again, and centrifuged at 11,337 × *g* for 1 min at RT. The resulting cell pellets were suspended in 2 ml or 200 µl of PBS (1×), depending if biofilms were grown on 24- or 96-well plates, respectively, and serial dilutions were cultured on TSA medium. The plated biofilm-encased and biofilm-free cells were incubated overnight, at 37°C, and CFUs per ml were counted.

### 5.3 Nitrite quantification

When appropriate, nitrite quantification was performed using the Griess colorimetric method (Tarpey et al., 2004) using freshly prepared solutions. Griess solution (sulfanilamide (1% m/v, Sigma, St. Louis, MO, USA), *N*-(1-naphthyl)ethylenediamine dihydrochloride (0.1% m/v, Sigma), and H_3_PO_4_ (2% v/v, Merck) in Milli-Q^®^ water) was mixed with the media recovered after cell culture in a 1:1 concentration. The absorbance at 450 nm was measured and fitted to a calibration curve obtained with known concentrations of NaNO_2_ (Merck).

### 5.4 *In vitro* experiments with silicone catheter

Silicone catheter pieces (1 or 2 cm) were incubated in 6-well polystyrene tissue culture plates containing 5 ml of DMEM+FBS that had been inoculated with *S. epidermidis* strains to an OD_600_ of approximately 0.05. After 2 h at 37°C, cells were exposed to 1 mM of DETANONOate and 5 mM of oxamate and incubated for 22 h. The images of the silicone sections were recorded with a digital camera. Then, the media were removed, and the plates were placed at 65°C for 2 h. The silicone pieces in the wells were submerged in 5 ml of 1% crystal violet for 20 min, washed and rinsed twice with PBS (1×), and air-dried. Images of the wells were acquired in an ImageScanner III LabScan 6.0 (600 dpi, GE Healthcare). The silicone tubes were then transferred to falcon tubes and destained by 10-min incubation with 5 ml of glacial acetic acid (33%), after which the CV absorption was measured at 590 nm.

### 5.5 Quantification of excreted metabolites by ^1^H-NMR

*S. epidermidis* strains were inoculated in DMEM plus FBS medium (or TSB, when indicated) to form biofilms, as described above. Supernatants (100 µl) were collected immediately after inoculation, after 24 and 48 h of growth, and centrifuged at 1,000 × *g* for 1 min. Then, 200 µl of cold (−80°C) liquid chromatography (LC)-grade methanol (Merck) was added, and the mixtures were incubated in dry ice for 30 min. To achieve complete FBS precipitation, samples were centrifuged at 16,000 × *g* for 20 min at 4°C. The resultant supernatants, containing the substrate and excreted metabolites (end-products), were placed under nitrogen flux for approximately 2 h until all the methanol was evaporated. The precipitates were stored at −20°C until further analysis. All precipitates were suspended in 600 µl of D_2_O, and samples were transferred to 5-mm NMR tubes for ^1^H-NMR analysis. The spectra were acquired in a Bruker Avance II 500-MHz spectrometer (Bruker BioSpin, GmbH, Ettlingen, Germany) operated by TOPSPIN software and using a 5-mm BBIXYZ high-resolution probe head at 16°C to allow the shift of the water peak from the glucose resonances, as described before (Carvalho et al., 2017; Carvalho et al., 2019). To acquire spectra, the standard Bruker pulse program zgprde was used for water presaturation with 12-µs pulse width and a relaxation delay time of 3.5 s (d1 = 2.5 s, d20 = 1 s) for saturated spectra or with an extra relaxation delay time (d20) of 60 s to allow full spin relaxation. Spectra were phase- and baseline-corrected and referenced to the resonance of externally added trimethylsilylpropanoic acid (TSP), which was assigned to 0 ppm. Metabolite concentrations were calculated from the areas of the resonances in ^1^H-NMR spectra by comparison to the area of the TSP resonance and after the application of an appropriate factor for correcting saturation of resonances and taking into account the number of protons, giving rise to the NMR signals/resonances.

### 5.6 Real-time RT-qPCR experiments

Biofilms were suspended in RNA Later (Sigma R0901) and PBS (1×) and stored at −20°C prior to RNA extraction. To isolate total RNA, biofilms were first centrifuged at 6,200 × *g*, 4°C, for 3 min to remove the RNA Later and suspended in 5% cold phenol RNA protective solution and 0.9% NaCl. Following centrifugation at 6,200 × *g* for 10 min at 4°C, the pellets were resuspended in 10 mM Tris–1 mM EDTA in DEPC water (pH 8). Lysozyme (12.5 mg/ml), mutanolysin (0.25 KU/ml), and proteinase K (2.5 mg/ml), prepared in nuclease-free water, were added to the buffered pellets, and the mixtures were incubated for 2 h at 37°C. Complete lysis was achieved by the addition of 350 µl of lysis buffer from the Aurum™ Total RNA Mini Kit (Bio-Rad, Hercules, CA, USA). For the highest purity, whenever complete lysis was not attained, samples were centrifuged 10 min at 4°C and 16,200 × *g* to remove non-lysed cells. After the addition of 250 µl of 70% isopropanol, the lysates were transferred to the Aurum RNA Binding Mini Columns, and total RNA was extracted using the respective kit and following the manufacturer’s instructions. RNA samples were concentrated by the NaAcet/EtOH method. In brief, 0.1 vol of 3 M sodium acetate (NaAcet), pH 5.5, and 2.5 volumes of pure cold ethanol were added to samples that were incubated at −80°C for 3 h. After this step, samples were thawed and centrifuged at 16,200 × *g* for 30 min at 4°C. RNA pellets were washed twice (16,200 × *g*, 10 min, 4°C) with 2.5 volumes of 75% ethanol and air-dried at RT for approximately 15 min, and RNAs were suspended in nuclease-free water. RNA samples were purified from contaminating DNA by treatment with the Ambion TURBO DNA-free™ DNase Kit (Invitrogen, Carlsbad, CA, USA/Thermo Fisher Scientific, Waltham, MA, USA). The absence of chromosomal DNA was confirmed by PCR using the oligonucleotides 5′-GCTAATGCCTCGTCAATAC-3′ and 5′-TATGGTGCTGGAACAGATAC-3′ for *S. epidermidis* 16S gene. RNA purity, integrity, and concentration were evaluated by agarose gel electrophoresis and using a NanoDrop 2000c UV-Visible spectrophotometer (Thermo Scientific).

The cDNA was obtained by reverse transcription using approximately 3 μg of total RNA and the Transcriptor High Fidelity cDNA Synthesis kit (Roche, Basel, Switzerland). Samples for real-time RT-qPCRs were prepared with reagents of the Light Cycler 480 SYBR Green I Master Kit, with the primers listed in **Table S2**, and reactions were performed in a Light Cycler 96 device (Roche). The expression ratios of the selected genes were normalized relative to the 16S rRNA gene of *S. epidermidis*, which does not change expression under the tested conditions. Ratios were calculated by the Livak and Schmittgen (Livak and Schmittgen, 2001) method.

### 5.7 Biofilm matrix composition analysis by confocal laser scanning microscopy

Biofilms were grown for 24 h as described above in 24-well µ-plates (82426, IBIDI^®^) to avoid background fluorescence in confocal microscopy. After removal of the media of each well, biofilms were stained with SYPRO Ruby (1×, FilmTracer™ SYPRO™ Ruby Biofilm Matrix stain, Invitrogen) and WGA (10 µg/ml, Wheat Germ Agglutinin, Oregon Green 488 Conjugate, Invitrogen) to label the biofilm matrix proteins and PNAG, respectively. The SYPRO Ruby/WGA mixture was incubated with the biofilms for 20  min in the dark, and the stained biofilms were washed one time with NaCl (0.9% m/v, Carl Roth, Karlsruhe, Germany). Confocal Z-series stacks were acquired in a Zeiss LSM 880 point scanning confocal microscope, using a 40× glycine immersion Plan-Apochromat lens (1.2 NA). Emission/excitation wavelengths were chosen according to fluorophores’ manufacturer instructions, and two separate virtual channels were used to avoid signal bleed-through. A minimum of three replicas per condition were performed, and in each well, five different-series images (73 × 73 µm) were taken in 0.5-µm slices. Image analysis was performed using BiofilmQ to determine fluorescence intensities and biofilm dimensions (Hartmann et al., 2021). The roughness of the biofilm matrices of individual confocal images of the biofilms was determined as $\frac{\text{mean }\left(d\right)}{N}\;.\sum_{i}^{N}\left|\text{mean }\left(d\right)-di\right|$ , with *N* denoting the total number of pillars and *di* the thickness of pillar I, as determined by BiofilmQ.

### 5.8 Transwell biofilm–macrophage co-culture assays

Murine macrophages J774A.1 (LGC Promochem, Teddington, UK) were cultured in DMEM (Gibco, ref. 31966021); supplemented with 10% (v/v) of FBS (Gibco, ref. 10270106), 50 U/ml of penicillin and 50 μg/ml of streptomycin (all reagents from Gibco, Amarillo, Texas, USA), and incubated in 5% CO_2_ atmosphere at 37°C. Macrophage cells per ml and viability were determined by resorting to an automatic cell counter (Countess 3, Invitrogen) and staining with 0.4% trypan blue (Sigma). Macrophages were seeded at 5 × 10^5^ cells/ml in wells of 24-well plates, incubated for 48 h, and then left inactivated or activated to the microbicidal M1 state by 5-h incubation with 5 μg/ml of lipopolysaccharide (LPS; Sigma) and 1 μg/ml of interferon-γ (IFN-γ; Sigma), in DMEM without antibiotics. Inhibition of iNOS was achieved by adding 800 µM of l-NMMA (*N* ^G^-monomethyl-l-arginine acetate salt, Sigma). *S. epidermidis* 1457 and RP62A were grown aerobically in TSB to an OD_600_ of 0.4–0.5, washed three times with PBS (1×), and resuspended in antibiotic-free DMEM medium to an initial bacterial OD_600_ of 0.05 (approximately 10^7^ cells/ml). After the 5-h incubation period for the activation of l-NMMA-exposed and l-NMMA-unexposed macrophages, macrophages were washed three times with PBS (1×), and bacteria were used to infect macrophages at a multiplicity of infection (MOI) of approximately 5, and after 30 min, the extracellular bacteria were eliminated by a 10-min treatment with DMEM supplemented with 50 µg/ml of gentamicin. For the controls without bacteria, macrophages were not only infected but also subjected to treatment with gentamicin. These macrophages were then washed three times with PBS (1×), and fresh DMEM devoid of antibiotics (1 ml), with and without 800 µM of l-NMMA, was added followed by 24-h incubation. The infected macrophages were washed three times with PBS (1×), put in contact with 0.4-μm-pore-sized inserts, and inoculated with 1457 or RP62A to an OD_600_ of 0.05. The volumes of antibiotic-free fresh DMEM, containing or lacking l-NMMA, added to the wells and inserts, were 600 and 400 µl, respectively. In this setup, the inserts allow the passage and diffusion of soluble components (e.g., nitric oxide) between macrophages present in the wells and bacteria in the inserts, but not the phagocytosis of bacterial cells (*S. epidermidis* cell size: 1–2 μm). After 24 h of growth, the inserts were collected and put in clean 24-well plates, and the amount of biofilm in the inserts was determined with the crystal violet assay as described above. The Griess method (Tarpey et al., 2004) was used to determine the nitrite present in macrophage supernatants, which results from the oxidation of the NO derived from iNOS activity.

## Data availability statement

The original contributions presented in the study are included in the article/**Supplementary Material**. Further inquiries can be directed to the corresponding author.

## Author contributions

SC and LS contributed to the conceptualization of this work and funding acquisition. SC and AO were involved in the methodology, investigation, formal analysis, visualization, and writing of the manuscript. SC was the project administrator, while LS performed a supervisor role and reviewed the manuscript. A few contents of this manuscript have been previously presented in the master thesis of the author AO. All authors contributed to the article and approved the submitted version.
