# Supplementary material for: Staphylococcus epidermidis biofilms undergo metabolic and matrix remodeling under nitrosative stress
Source: Front Cell Infect Microbiol. 2023 Jul 4;13:1200923. doi: 10.3389/fcimb.2023.1200923 (PMC10352803; doi:10.3389/fcimb.2023.1200923)
Supplement: Supplementary file 1 [file DataSheet_1.pdf]

Supplementary Material

Staphylococcus epidermidis biofilms undergo metabolic and matrix remodeling under nitrosative stress

Ana S Oliveira, Lígia M Saraiva, and Sandra M Carvalho*

Instituto de Tecnologia Química e Biológica António Xavier, Universidade Nova de Lisboa (ITQB NOVA), Oeiras Portugal

Running title: Adaptive response of *S. epidermidis* biofilms to NO

*** Correspondence:**
Corresponding Author
smcc@itqb.unl.pt

Keywords: ***Staphylococcus epidermidis*, biofilm metabolism, nuclear magnetic resonance (NMR), confocal laser scanning microscopy (CLSM), nitrosative stress**

Supplemental Tables

Table S1. *Staphylococcus epidermidis* strains used in this study

S. epidermidis	**Description**	**Source**
**1457**	Clinical isolate from a central venous catheter infection, methicillin-sensitive, biofilm (+) and *icaADBC* (PIA) (+)	Rohde, H. University of Hamburg, Germany
**RP62A**	Clinical isolate from an intravascular catheter-associated infection, methicillin-resistant, biofilm (+) and *icaADBC* (PIA) (+)	Götz, F. University of Tübingen, Germany
**M12**	1457Δ*yabJ-spoVG*::Tn917 mutant, described as PIA defective; Erm^R	Rohde, H. University of Hamburg, Germany

Abbreviations: Erm^R, erythromycin resistance.

Table S2. Primers used in RT-qPCR assays

Primer name	Sequence (from 5' to 3')
***16S*_fw_RT**	GCGAAGAACCTTACCAAATC
***16S*_rev_RT**	CCAACATCTCACGACACG
***ldh*_fw_RT**	GTGCTTGATAGTGCAAGATTC
***ldh*_rev_RT**	GATATCGTAAGCAGCATCTCT
***glmM*_fw_RT**	GGTGCACAAAAGTATCTAAGTTATC
***glmM*_rev_RT**	GAGTAGAGCCACATTGTTCATT
***glmU*_fw_RT**	GGTGTTAATGATCGTTTGATG
***glmU*_rev_RT**	AGAGTATTGGCCTATCCACAC
***icaA*_fw_RT**	GCAAGTATTATTGGTTGTATCAAG
***icaA*_rev_RT**	CACTAACATCCAGCATAGAGC

SUPPLEMENTAL FIGURES

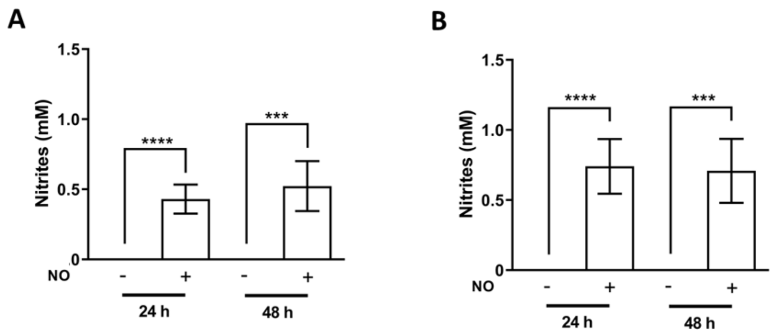

Figure S1. Nitrite accumulation of *S. epidermidis* biofilms exposed to NO. *S. epidermidis* biofilms of strains 1457 **(A)** and RP62A **(B)**, grown for 24 and 48 h without and with 1 mM NO, were evaluated by measuring the extracellular nitrite accumulation. Error bars represent mean ± SD (n ≥ 6). Welch's t-tests were performed. Asterisks represent statistically significant data relative to control (****, $p \leq 0.0001$; ***, $p \leq 0.001$).

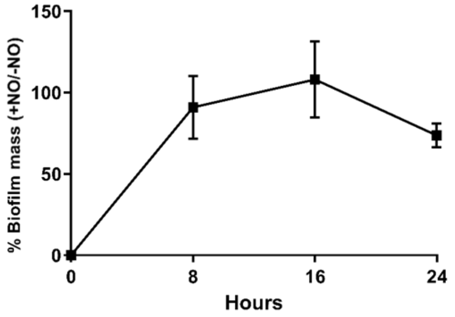

Figure S2. Biofilm formation over time under NO exposure. Amount of biofilm formed by *S. epidermidis* 1457 exposed to 1 mM NO relative to the untreated biofilm. Error bars represent mean ± SD (n = 3).

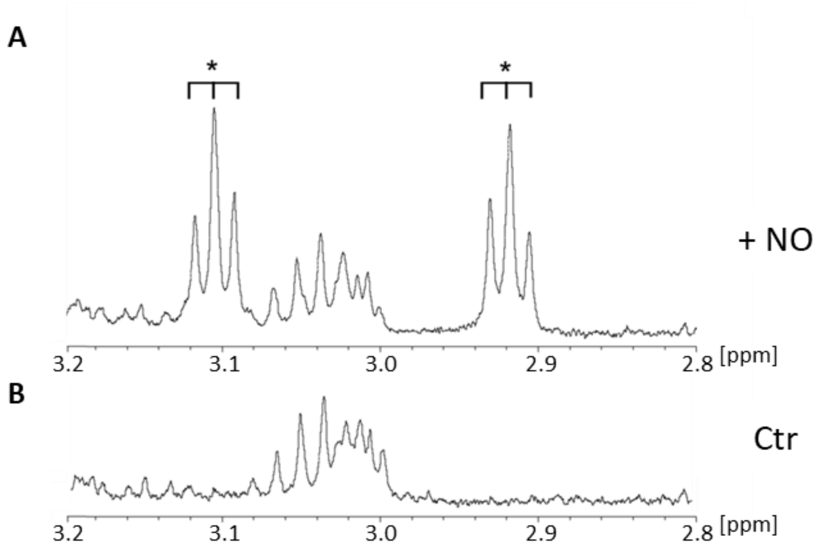

Figure S3. ^{1}H-NMR spectra of supernatants of *S. epidermidis* biofilms grown in high glucose DMEM/FBS for 24 h, in the presence **(A)** and in the absence **(B)** of 1 mM NO. Figure shows the 2.8 - 3.2 ppm region from representative experiments of 1457 and RP62A strains. NO-exposed biofilms excrete an unknown compound that appears as a doublet of triplets, and is marked with an asterisk (*).

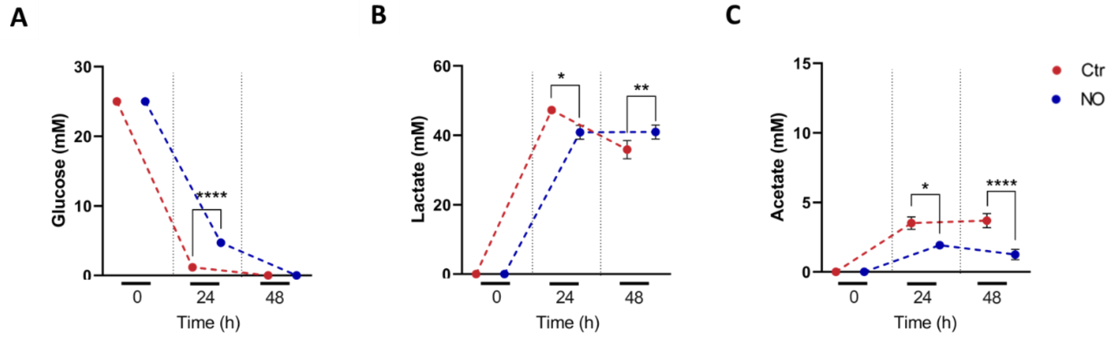

Figure S4. Substrate consumption and major end-products formed by NO-exposed *S. epidermidis* M12. Glucose consumption **(A)** and extracellular metabolites: lactate **(B)**, and acetate **(C)** that accumulated in biofilms of *S. epidermidis* M12 grown in high glucose DMEM/FBS medium in the absence (Ctr, red dots) and presence of 1 mM NO (blue dots). Substrate and end-product quantification was performed on biofilm supernatants by ^{1}H-NMR at 0, 24 and 48 h. To allow better observation of the differences between the conditions, the points at each hour in each graph were represented with a small shift, which does not indicate measurements at different times. Error bars represent concentrations means ± SD (n = 3 for 24 h and n = 6 for 48 h). All comparisons were performed using Welch's t-tests. Asterisks represent statistically significant data relative to control (****, $p \leq 0.0001$; **, $p \leq 0.01$; *, $p \leq 0.05$).

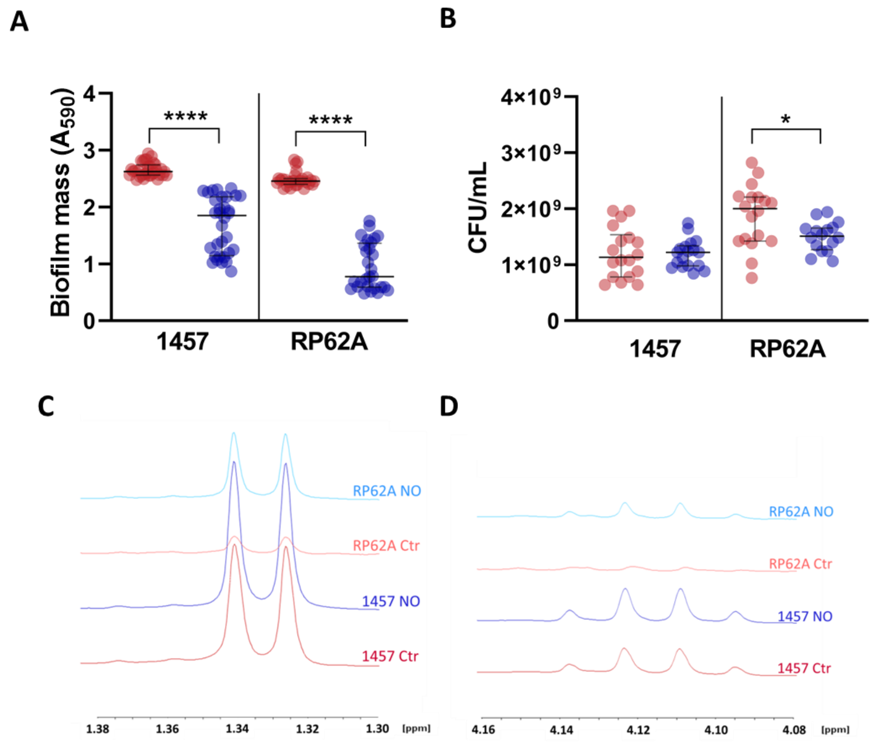

Figure S5. Biofilm amount, viability and extracellular lactate amount in *S. epidermidis* biofilms grown in TSB and exposed to NO. Amount of biofilm **(A)** and viable cells **(B)** produced by *S. epidermidis* 1457 and RP62A after 48 h of growth in TSB media in the absence (Ctr, red dots) and presence of 1 mM NO (blue dots). Scattered symbols represent individual measurements ($n \geq 18$) and horizontal lines indicate median values and interquartile range. Comparisons were performed using Welch's t-tests. Asterisks represent statistically significant data relative to control (****, $p \leq 0.0001$; * $p \leq 0.05$). ^{1}H-NMR spectra of supernatants of biofilms of *S. epidermidis* 1457 (dark blue and red) and RP62A (light blue and red) grown in TSB for 48 h, in the presence of 1 mM NO (blue) and in the absence (red). The figure shows the lactate doublet in **(C)** and the quartet in **(D)**. The figure is a representative experiment of four independent assays.

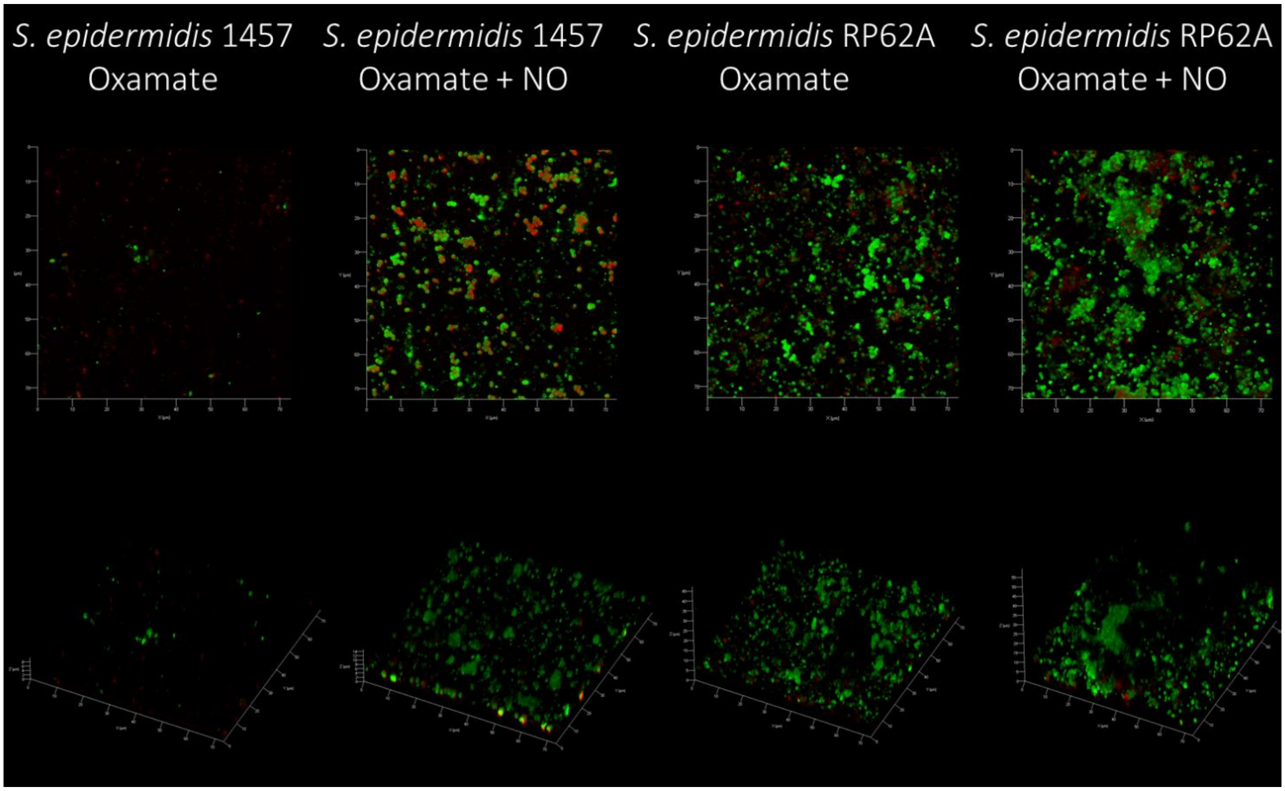

Figure S6. Amount of matrix polymers in biofilms of *S. epidermidis* exposed to oxamate and NO. Confocal images, depicted as Z and orthogonal projections, representative of the *S. epidermidis* 1457 and RP62A biofilms grown with 5 mM oxamate, exposed or unexposed (control) to 1 mM NO, with matrix proteins and polysaccharides in red and green, respectively. Each image shows a 75 X 75 µm section of the biofilm with varying heights. Each component was detected by staining with appropriate fluorophores.

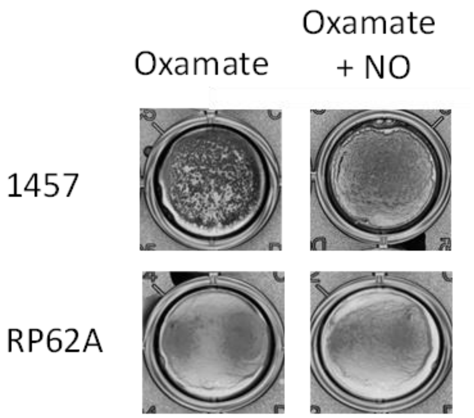

Figure S7. Macroscopic images of *S. epidermidis* biofilms treated with oxamate and NO. *S. epidermidis* 1457 and RP62A biofilms grown for 24 h in DMEM+FBS medium in the presence of 5 mM oxamate or 5 mM oxamate plus 1 mM NO (Oxamate + NO), and stained with crystal violet.

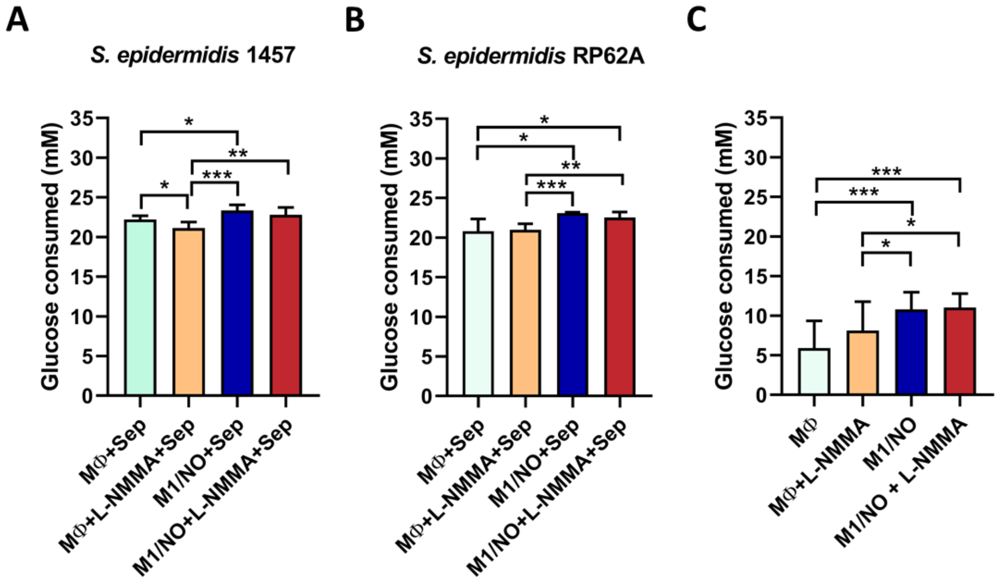

Figure S8. Glucose consumption by monocultures of macrophages, and macrophages co-cultivated with *S. epidermidis* biofilms. Glucose consumed after 24 h from the supernatants of L-NMMA-untreated (dark and light blue bars) and -treated (orange and red bars), M1-activated (dark blue and red bars) and MØ non-activated (light blue and orange bars) macrophages, co-cultivated with *S. epidermidis* strains 1457 **(A)** and RP62A **(B)** in inserts/transwells in high glucose DMEM/FBS medium and cultivated alone (monocultures of MØ and M1) **(C)**. Glucose concentrations were determined by ^{1}H-NMR. Error bars represent mean ± SD (n = 6). Comparison was performed using Welch's t-test.
